# Platinum-resistance in epithelial ovarian cancer: an interplay of epithelial–mesenchymal transition interlinked with reprogrammed metabolism

**DOI:** 10.1186/s12967-022-03776-y

**Published:** 2022-12-03

**Authors:** Dilys Leung, Zoe K. Price, Noor A. Lokman, Wanqi Wang, Lizamarie Goonetilleke, Elif Kadife, Martin K. Oehler, Carmela Ricciardelli, George Kannourakis, Nuzhat Ahmed

**Affiliations:** 1 Fiona Elsey Cancer Research Institute, Ballarat Central Technology Central Park, Ballarat, Vic 3353 Australia; 2 Discipline of Obstetrics and Gynaecology, Adelaide Medical School, Robinson Research Institute, The University of Adelaide, Adelaide, SA 5005 Australia; 3 Department of Gynecological Oncology, Royal Adelaide Hospital, Adelaide, SA 5000 Australia; 4 School of Science, Psychology and Sport, Federation University, Mt Helen, VIC 3350 Australia; 5 Department of Obstetrics and Gynaecology, University of Melbourne, Melbourne, VIC 3052 Australia; 6 Centre for Reproductive Health, Hudson Institute of Medical Research and Department of Translational Medicine, Monash University, Clayton, VIC 3168 Australia

**Keywords:** Epithelial–mesenchymal transition, Epithelial ovarian cancer, AKR1B1, ITGAV, TGFB1, Platinum-resistance

## Abstract

**Background:**

Epithelial ovarian cancer is the most lethal gynaecological cancer worldwide. Chemotherapy resistance represents a significant clinical challenge and is the main reason for poor ovarian cancer prognosis. We identified novel expression of markers related to epithelial mesenchymal transitions (EMT) in a carboplatin resistant ovarian cancer cell line by proteomics. This was validated in the platinum resistant versus sensitive parental cell lines, as well as platinum resistant versus sensitive human ovarian cancer patient samples. The prognostic significance of the different proteomics-identified marker proteins in prognosis prediction on survival as well as their correlative association and influence on immune cell infiltration was determined by public domain data bases.

**Methods:**

We explored the proteomic differences between carboplatin-sensitive OVCAR5 cells (parental) and their carboplatin-resistant counterpart, OVCAR5 CBPR cells. qPCR and western blots were performed to validate differentially expressed proteins at the mRNA and protein levels, respectively. Association of the identified proteins with epithelial–mesenchymal transition (EMT) prompted the investigation of cell motility. Cellular bioenergetics and proliferation were studied to delineate any biological adaptations that facilitate cancer progression. Expression of differentially expressed proteins was assessed in ovarian tumors obtained from platinum-sensitive (n = 15) versus platinum-resistant patients (n = 10), as well as matching tumors from patients at initial diagnosis and following relapse (n = 4). Kaplan–Meier plotter and Tumor Immune Estimation Resource (TIMER) databases were used to determine the prognostic significance and influence of the different proteomics-identified proteins on immune cell infiltration in the tumor microenvironment (TME).

**Results:**

Our proteomics study identified 2422 proteins in both cell lines. Of these, 18 proteins were upregulated and 14 were downregulated by ≥ twofold (*p* < 0.05) in OVCAR5 CBPR cells. Gene ontology enrichment analysis amongst upregulated proteins revealed an overrepresentation of biological processes consistent with EMT in the resistant cell line. Enhanced mRNA and/or protein expression of the identified EMT modulators including *ITGA2*, *TGFBI*, *AKR1B1*, *ITGAV*, *ITGA1*, *GFPT2*, *FLNA* and *G6PD* were confirmed in OVCAR5 CBPR cells compared to parental OVCAR5 cell line. Consistent with the altered EMT profile, the OVCAR5 CBPR cells demonstrated enhanced migration and reduced proliferation, glycolysis, and oxidative phosphorylation. The upregulation of G6PD, AKR1B1, ITGAV, and TGFβ1 in OVCAR5 CBPR cells was also identified in the tumors of platinum-resistant compared to platinum-sensitive high grade serous ovarian cancer (HGSOC) patients. Matching tumors of relapsed versus newly diagnosed HGSOC patients also showed enhanced expression of AKR1B1, ITGAV, TGFβ1 and G6PD protein in relapsed tumors. Among the identified proteins, significant enhanced expression of GFPT2, FLNA, TGFBI (CDGG1), ITGA2 predicted unfavorable prognosis in ovarian cancer patients. Further analysis suggested that the expression of TGFBI to correlate positively with the expression of identified and validated proteins such as GFPT2, FLNA, G6PD, ITGAV, ITGA1 and ITGA2; and with the infiltration of CD8^+^ T cells, macrophages, neutrophils, and dendritic cells in the TME.

**Conclusions:**

Our research demonstrates proteomic-based discovery of novel EMT-related markers with an altered metabolic profile in platinum-resistant versus sensitive ovarian cancer cell lines. The study also confirms the expression of selected identified markers in the tumors of platinum-resistant versus sensitive, and in matching relapsed versus newly diagnosed HGSOC patients. The study provides insights into the metabolic adaptation of EMT-induced carboplatin resistant cells that confers on them reduced proliferation to provide effective migratory advantage; and the role of some of these identified proteins in ovarian cancer prognosis. These observations warrant further investigation of these novel target proteins in platinum-resistant patients.

**Supplementary Information:**

The online version contains supplementary material available at 10.1186/s12967-022-03776-y.

## Introduction

Ovarian cancer is a heterogeneous disease that is categorized historically as a malignancy derived through the transformation of epithelial, sex-cord stromal or germ cells. Ninety percent of all cases are epithelial tumors, of which the most common serous subtype constitutes 80% of the cancer [1]. Surgery is the first line of treatment for ovarian cancer patients, while platinum- and taxane-based combination chemotherapy is given as a standard of care following surgery. However, the majority of ovarian cancer patients eventually experience a relapse due to failure of chemotherapy treatments, resulting in 30–40% 5-year survival rate, which has remained stagnant for the last three decades.

Platinum resistance is the major challenge in ovarian cancer treatment. The current classification strictly defines resistance to platinum as recurrence within 6 months of the last platinum administration [2]. Duration between the last administration of platinum treatment and cancer relapse dictates future line of treatment strategies with either platinum-based or another chemotherapeutics in the clinic. However, some argue that the empiric 6-month cut-off criterion for platinum-resistance may not be biologically relevant. More work needs to be done to identify mechanisms and molecular markers, which can be used clinically to design more effective targeted treatments for ovarian cancer patients [2].

Epithelial mesenchymal transition (EMT) has been associated with the acquisition of migratory and invasive like properties in cancer cells [3, 4]. During EMT, transcriptional and metabolic reprogramming triggers morphological and functional changes in cancer cells [5]. Loss of E-cadherin (CDH1) expression with the gain in N-cadherin and vimentin (VIM) expression associated with mesenchymal phenotype (enhanced migration, secretion of extracellular matrix [ECM] degrading proteases and ECM remodeling etc.) have been clinically associated with poor prognosis in many cancers [3, 6–8]. In recent years, a good thrust of studies, including those in ovarian cancer, have demonstrated ‘incomplete or hybrid EMT’ or transitory cells within a ‘spectrum of EMT’ in tumors or cell lines where they retain both epithelial and mesenchymal features, and are able to migrate and invade like in a classical EMT process [6, 9]. Recent literature also suggests that EMT transformed cells can endorse on themselves the ability to evade host immunity by initiating several mechanisms. These include alterations in the antigen-processing machinery such as downregulation of MHC Class 1 molecule or transporters associated with antigen processing (TAP-1 and TAP-2) or enhancement in the expression of check point molecules such as PD-L1 on cancer cells to inhibit T cell function. In addition, genetic alterations which interfere with the interferon-gamma (IFN-γ) signaling pathway are also known to affect the antitumor responses in melanoma patients treated with immunotherapy [10].

An association between chemoresistance and the acquisition of EMT in ovarian cancer cells that attain cancer stem cells (CSC)-like phenotype has been demonstrated [11–13]. These are a subpopulation of cells that possess the capacity of self-renewal and are responsible for drug resistance, tumor relapse and progression. They exploit therapy-induced selection pressure to give rise to resistant clones through plastic mechanisms such as EMT, which results in altered gene and protein expression and composition. Activation of EMT reprogramming and facilitation of cancer metastasis has been reported in multiple cancers through the induction of TGFβ and its inducible secreted extracellular matrix (ECM) associated proteins [14–18]. The role of TGFBI is dependent on cellular context as its expression is elevated or suppressed in many cancers [19]. High expression of TGFBI interlinked with poor prognosis in patients has been noted in muscle invasive bladder cancer compared to non-muscle invasive bladder cancer tissues [15], pancreatic cancer [20], colorectal cancer [14], etc.

In addition to the tumor biology roles of TGFBI, pan-cancer analysis has indicated a prognostic role of TGFBI and associated that with various immune responses and functions (https://papers.ssrn.com/sol3/cf_dev/AbsByAuth.cfm?per_id=4710390). In pancreatic cancer, cancer-associated fibroblasts express high levels of TGFBI which directly acts on tumor-specific CD8^+^ T cells and F4/80 macrophages in mice, reducing their proliferation and activation [20]. Targeting TGFBI in established lesions functionally reprogrammed F4/80 macrophages in tumor microenvironment [20]. In ovarian cancer, secreted TGFBI from tumor-associated macrophages in the ascites of ovarian cancer patients have been shown to promote migration of tumor cells [21, 22]. These observations suggest that tumor associated TGFBI is not only critical for driving cancer progression and metastasis but is also central in regulating cancer associated immune responses in host.

To understand the molecular basis of drug resistance in these cells, we analyzed the proteome in ovarian cancer cells and their platinum resistant counterpart in vitro. Our proteomic analyses revealed an enhanced expression of EMT and metabolic modulators in the carboplatin-resistant ovarian cancer cells. Given the increased expression of EMT modulators in our carboplatin-resistant ovarian cancer cells, we sought to explore this further by in vitro migration and bioenergetic assays. Evaluation of selected key proteomics-identified EMT-related proteins in platinum-sensitive, resistant, newly diagnosed, and relapsed ovarian tumor samples from ovarian cancer patients showed enhanced expression of some of these EMT-associated proteins in platinum-resistant vs sensitive and relapsed versus newly diagnosed patient’s tumors. Analysis with Kaplan–Meier plotter correlated high expression of some of the identified proteins as negative prognostic indicators. In addition, TIMER database analysis showed correlative positive expression of TGFBI with some of the identified proteins and their effect on infiltrating immune cells within the TME. Using these different platforms, our study provided a unified result depicting an induced EMT with an altered metabolic reprogramming in platinum-resistant ovarian cancer cells. This study has the potential to open avenues to design therapeutics aimed at targeting specific EMT- and metabolism-associated proteins to circumvent platinum resistance in ovarian cancer.

## Methods

### Cell lines

The OVCAR5 parental and carboplatin resistant (CBPR) counterparts were developed in Dr Ricciardelli’s laboratory [23]. Cells were maintained in Gibco™ RPMI 1640 media (Thermo Fisher Scientific, Waltham, MA, U.S.A.) supplemented with 10% (v/v) fetal bovine serum (FBS) (Thermo Fisher Scientific) at 37 °C in a 95% air/5% CO_2_ humidified incubator.

### MTT assay

Cells (5000 cells/well) were plated in 96-well plates in RPMI growth medium. After 24 h, cells were treated with increasing concentrations of carboplatin (Hospira Pty Ltd, 5–200 µM). Cell survival was assessed by MTT assay after 72 h as per manufacturer’s instructions (Sigma Aldrich). Absorbance was read at 595 nm on a microplate absorbance reader (Triad series multimode detector, Dynex technologies, Chantilly, VA, USA). GraphPad Prism (version 8.0.0) was used to calculate the IC_50_ of carboplatin in both OVCAR5 and OVCAR5 CBPR cells.

For proliferation assay, serial dilution of OVCAR5 parental and OVCAR5 CBPR cells was prepared and seeded at a density ranging from 5 × 10^2^ to 1 × 10^5^ per well in a 96-well plate and incubated overnight. Tetrazolium salt from the Cell Proliferation Kit I (MTT) (Roche Diagnostics, Risch-Rotkreuz, Switzerland) was then added to the wells at a final concentration of 0.5 mg/ml. The microplate was incubated for 4 h before 10 μl of solubilization solution was added into each well to aid complete solubilization of formazan crystals produced by metabolically active cells. Following an overnight incubation, the absorbance reading was taken at 595 nm on an iMark™ microplate absorbance reader (Bio-Rad Laboratories, Hercules, CA, USA).

### IncuCyte proliferation assay

OVCAR5 parental and OVCAR5 CBPR cells were seeded at 7.5 × 10^3^ cells/well in 96 well plates. Whole well images were taken every 2 h from 0 to 48 h using IncuCyte^®^S3 Live Cell Analysis system (Sartorius, Ann Arbor, MI). Cell confluency was calculated using IncuCyte^®^Base Analysis Software.

### Mass spectrometry analysis

#### Sample preparation

Control experiment using total protein lysates of OVCAR5 parental cells (triplicate samples) was first performed to demonstrate technical reproducibility of the protocol described below. Total protein lysates in Pierce^®^RIPA buffer (supplemented with protease inhibitor cocktail tablet; Thermo Fisher Scientific) were collected from 3 passages of OVCAR5 parental and OVCAR5 CBPR cells and were processed using the filter aided sample preparation (FASP) method with minor adjustments.

All centrifugations were performed at 14,000 ×*g* for 15 min at room temperature unless stated otherwise. All chemicals are HPLC grade purchased from Sigma-Aldrich unless otherwise stated. Protein quantification was performed using Pierce™ BCA protein assay kit (Thermo Fisher Scientific). 40 μg of total protein was mixed with 100 μl of 8 M urea/50 mM TEAB and then loaded into centrifugal filter unit (molecular weight cut-off = 30 kDa; Merck, Kenilworth, NJ, USA), followed by centrifugation. The filter unit was washed with 200 μl of 8 M urea/50 mM TEAB and was centrifuged. The sample was then incubated with 10 mM TCEP at 37 °C with shaking for 30 min. Flow-through was discarded following centrifugation. Incubation with iodoacetamine (55 mM) was performed in the dark at 37 °C for 45 min with shaking. Following centrifugation, the filter unit was washed twice with 100 μl 8 M urea/50 mM TEAB. 100 μl of 0.05 M NH_4_HCO_3_ was added to the membrane twice, each followed by 10-min centrifugation; flow-through was discarded. Proteins were digested with trypsin/Lys-C (trypsin/Lys-C:protein = 1:40) (Promega, Madison, Wisconsin, USA) overnight at 37 °C. 40 μl 0.05 M NH_4_HCO_3_ was added and the filter unit was centrifuged. The flow-through containing digested peptides was acidified to 1% TFA prior to LC–MS analysis using an Orbitrap Elite™ hybrid ion trap-orbitrap mass spectrometer (Thermo Fisher Scientific) at the Bio21 Mass Spectrometry and Proteomics Facility, University of Melbourne.

#### MS data processing

All.RAW files were processed using MaxQuant (version 1.6.2.3) and its built-in Andromeda search engine with orbitrap selected as instrument. The corresponding default parameters were used unless otherwise stated. Peptide and protein false discovery rate were both set at 1%. Match between runs was enabled. Label-free quantification (LFQ) intensity profiles were determined with a minimum ratio count of 2. Peptide sequences were mapped against human protein database (Swiss-Prot, *Homo sapiens*, canonical, May 2019, 20365 entries).

#### Bioinformatics workflow

Perseus software (version 1.5.3.1) was utilized to filter the main data matrix. Identifications from the reverse decoy database, identified by site only, and those with razor and unique peptides < 1 were excluded. Technical reproducibility of the control experiment was confirmed using multiple regression analysis (R^2^ > 0.97) and visualized on multi scatter plot.

Fold changes were calculated using the average of three LFQ intensity values of each identified protein in OVCAR5 parental and OVCAR5 CBPR samples. Unique proteins or differentially expressed proteins at significant level (*p* < 0.05 and ≥ twofold) were included for further analysis. STRING software (*Search Tool for the Retrieval of Interacting Genes/Proteins*; version 11) was used to visualize protein network and compute functional enrichment analysis for GO (gene ontology) biological process (*Homo sapiens*)[24].

### RNA isolation and real-time reverse transcription polymerase chain reaction (RT–qPCR)

RNA was isolated using RNasy^®^plus mini kit (Qiagen, Hilden, Germany) or the TaqMan^®^Gene expression Cells-to-CT™ kit (Applied Biosystems, Mulgrave, Victoria, Australia), according to manufacturer’s instructions. RNA quality and concentration were analyzed using a NanoDrop 1000 spectrophotometer (Thermo Fisher Scientific). RT2 first strand kit (Qiagen) was used to remove any contaminating genomic DNA and synthesize cDNA with 1 μg RNA as per manufacturer’s instructions.

qPCR was performed using RT2 SYBR green master mix (Qiagen) run on a Rotor-Gene Q real-time cycler (Qiagen). Each 20μl reaction mix contains 10μl of RT2 SYBR green master mix, 0.8μl of each of forward and reverse primers (10 μM), 1μl of cDNA and 7.4μl of RNase-free H_2_O. qPCR parameters were as follow: 95 °C for 10 min, 45 cycles of 95 °C for 15 s and primer-specific annealing temperature for 30 s. Primer sequences are listed in Additional file 1: Table S1.

qRT–PCR reactions were performed using TaqMan^®^primer sets for *CD44* (Hs01075864_ml), *ABCG2* (Hs01053790_ml), VIM (Hs00185584_ml), *G6PD* (Hs00959070_ml), *CDH1* (Hs00170423_ml) and the primer sets described in Additional file 1: Table S1, using the Quantsudio 12 K Flex Real Time PCR System (Applied Biosystems). PCR cycling conditions were as follows: 50 °C for 2 min, 95 °C for 10 min (with 40 cycles following 95 °C for 15 s), and 60 °C for 1 min. CT values were normalized to the house keeping gene β-actin (Human *ACTB* 4333762, Applied Biosystems) or 18S genes and calibrated using the 2^−∆∆CT^ method. All reactions were run three times in triplicates.

### Western blot analysis

30μG of total protein was separated by SDS-PAGE gel (4–20% resolving; Bio-Rad Laboratories) and transferred to a PDVF membrane. Non-specific binding was blocked by 5% BSA in tris-buffered saline for 1 h at room temperature prior to overnight incubation with primary antibody at 4 °C. Membrane was then washed and incubated with horseradish peroxidase-conjugated anti-mouse IgG (1:4000; Cell Signaling Technologies, Danvers, MA, USA, #7076), or goat anti-rabbit secondary antibody (1:4000; Bio-Rad Laboratories, #1706515) for 2 h at room temperature. Protein bands were visualized using the enhanced chemiluminescence reagents (Bio-Rad Laboratories). Quantification by densitometry with Image Lab™ software (Bio-Rad Laboratories) was performed using β-actin as internal control. To probe for respective proteins, we used anti-ITGAV (1:2500; Abcam, Cambridge, UK; ab179475), anti-AKR1B1 (1:1000; Abcam, ab175394), anti-TGFβ1 (1:1000; Abcam, ab190503), anti-G6PD (1:5000, Abcam, ab993) and anti-actin (1:1000; Cell Signaling Technologies, #8457) antibodies.

### G6PD activity assay

G6PD activity was measured in cell extracts of OVCAR5 parental and OVCAR5 CBPR (2.0 × 10^6^ cells) cells using a colorimetric based assay (MAK015, Sigma Aldrich). Cell pellets was resuspended in 50µL of PBS and diluted 1/10 in assay buffer. All standards and positive controls were prepared according to the manufacturer’s instructions. Absorbance values were measured at 450 nm using Triad series multimode detector after 15 min (Dynex technologies, USA).

### Wound healing assay

OVCAR5 parental and OVCAR5 CBPR cells were seeded at 5 × 10^5^ /well and maintained under normal culture condition 24 h. On the day of assay, a p1000 tip was used to create a cell-free area. Cells were then washed with PBS and cultured in RPMI containing 2% FBS to suppress proliferation. Images of wound were taken between 0 and 24 h using IncuCyte^®^S3 Live Cell Analysis system (Sartorius, Ann Arbor, MI). Wound area was assessed using FIJI software and the Wound_healing_size_tool plug in (ImageJ, NIH, Version:2.0.0-rc-69/1.52n) in matching areas at 0 and 9 h (7 areas/well). Difference in wound area was calculated for each area and averaged for each well.

### Immunofluorescence

OVCAR5 parental and OVCAR5 CBPR cells were seeded at 2 × 10^4^ cells/well in 8 well tissue culture chamber slides (Nunclon™ Lab-Tek II Chamber slide, RS Glass Slide, Naperville, IL). Cells were cultured for 48 h and washed with PBS before fixation in 4% paraformaldehyde for 10 min followed by 5 min in ice cold methanol. Cells were washed in PBS before blocking in 5% goat serum and overnight incubation with vimentin antibody (1/250, GeneTex, GTX100619). Cells were washed in PBS and incubated 1 h at room temperature with anti-rabbit IgG (H + L) Alexa FluorTM Plus 594 (1/400, A32740, Invitrogen,). Nuclei were stained with DAPI (1.5 µg/mL, Molecular Probes, Life Technologies) at room temperature for 15 min, and slides mounted with Prolong Gold Antifade Mountant with DAPI (# P36941, Molecular Probes, Life Technologies). Cells were imaged at 40X objective using the BX50 epifluorescence microscope (Olympus, Australia). Intensity density of vimentin staining in individual cells was quantified using FIJI (ImageJ, NIH, Version:2.0.0-rc-69/1.52n).

### Extracellular flux assay

Cellular bioenergetic profiling of the OVCAR5 parental and CBPR cells was assessed using the glycolysis stress kit (Agilent Technologies, Santa Clara, CA, U.S.A.) on a Seahorse Extracellular Flux XFp Analyzer (Agilent Technologies) according to manufacturer’s instruction. Briefly, the sensor cartridge was hydrated with calibrant overnight prior to cell seeding. 3 × 10^4^ OVCAR5 parental or CBPR cells were plated in triplicate wells, with 2 additional wells containing media only for background correction. 24 h post-seeding, cells were washed twice with the XFp base medium (supplemented with 2 mM glutamine; pH 7.4). Compounds including glucose (10 mM), oligomycin (1.5 μM), and 2-DG (50 mM) were reconstituted using XFp base medium. These were sequentially injected to the wells over the course of the assay as per the default template in the analyzer. Raw data were normalized against total protein amount measured using the Pierce BCA protein assay (Thermo Fisher Scientific) upon assay completion. Data analysis was performed with the analyzer’s Wave software (version 2.6.0.31).

### Human ovarian tumor samples

Ovarian tumor samples were collected with patient consent and approval by the Royal Adelaide Hospital Human Ethics Committee (RAH protocols #060,903 and #140,201). Patients were classified as platinum sensitive if they exhibited a complete response and did not progress within 6 months after completing the chemotherapy treatment. Patients were classified as platinum resistant if they did not respond to chemotherapy treatment or relapsed within 6 months of treatment. Clinical information of patients is listed in Additional file 2: Table S2.

Immunohistochemistry was performed on tissue sections as described previously [23]. Tissue sections were blocked with 5% goat serum (30 min) and incubated overnight at 4 °C with primary antibodies: G6PD (1:800, rabbit polyclonal, ab993, Abcam), AKR1B1 (1:250, rabbit polyclonal, ab175394, Abcam), TGFbeta1 (1:250, mouse monoclonal, clone TB21, ab190503, Abcam) and ITGAV (1:600, rabbit polyclonal, ab179475, Abcam). Tissue sections were subsequently incubated sequentially with secondary antibodies: biotinylated goat anti-rabbit (1:400, Dako, Australia) or biotinylated goat anti-mouse (1:400, Dako, Australia) followed by streptavidin–horseradish peroxidase (1:500, Dako, Australia) at room temperature (1 h). Peroxidase activity was detected using diaminobenzidine (DAB) and H_2_O_2_ (Sigma-Aldrich). Sections were counterstained with haematoxylin (Sigma-Aldrich), dehydrated with 70% and 100% ethanol and xylene and mounted in Pertex (Medite Medizintechnik, Germany). Tissues without primary antibody or with compatible mouse/immunoglobulins were included as negative controls. Human placenta tissue was used as a positive control tissue.

### Kaplan–Meier plot

Database comprising gene expression data and survival information of ovarian cancer patients from Gene Expression Omnibus and The Cancer Genome Atlas (TCGA) was utilized to explore the prognostic value of few EMT and metabolic modulators described in the study [25]. Expression of these genes and their association with progression-free survival were explored in gene expression dataset of ovarian cancer patients. Publicly available gene expression dataset of high-grade human ovarian cancers including GSE14764, GSE15622, GSE26193, GSE30161, GSE63885, GSE9891 and TCGA, were used for analysis. Analysis was restricted to patients with advanced stage (stage 3 and 4) high-grade serous ovarian cancers. Information on variants of TP53 including both mutant and wild type, debulking procedures and history of chemotherapies were included. Final cohort for analysis consists of 738 patients when biased arrays were excluded.

### TIMER database

TIMER (https://cistrome.shinyapps.io/timer) web server contains gene expression profiling of 10,897 cancer samples covering 32 different TCGA-derived cancers. It provides a comprehensive computational method to analyze cancer-related genes and infiltration of immune cell subtypes across diverse range of cancers. The abundances of six immune infiltrates (B cells, CD4^+^ T cells, CD8^+^ T cells, neutrophils, macrophages, and dendritic cells) are estimated by TIMER algorithm. The TIMER web server was used to develop scatter plots indicating associations between different proteomics identified proteins and their effect on the infiltration of different sub-sets of immune cells in TME.

### Statistical analyses

Biological assays including qRT–PCR, western blot, extracellular flux assays and migration assays were performed in triplicate in three independent experiments. Data are presented as mean ± SD. Comparisons between chemo-sensitive and -resistant groups were performed using student’s *t*-test or the non-parametric Mann-Whitney or Wilcoxon matched-pairs signed rank test unless stated otherwise; *p*<0.05 is considered statistically significant.

## Results

To understand the molecular changes between parental and carboplatin-resistant cells, we analyzed the proteomic changes in OVCAR5 parental and its carboplatin-resistant counterparts, OVCAR5 CBPR cell lines. Altogether, 2579 and 2576 proteins were identified in OVCAR5 parental and CBPR cell lines, respectively. Following filtering strategies described above, 2422 proteins were subjected to subsequent analyses. To analyze significant differences between OVCAR5 parental and CBPR cells, a selection criterion incorporating proteins which exhibited a fold change of > 2 at a statistically significant level (*p* < 0.05) were included in the study. Of these, 18 were upregulated (Table 1) and 14 downregulated (Table 2) by ≥ twofold at significant level (*p* < 0.05) in OVCAR5 CBPR cell lines. The peptide profile of up- and down-regulated proteins in OVCAR5 CBPR compared to OVCAR5 parental cell line is described in Additional file 2: Tables S3A and B.

**Table 1:** Proteins upregulated in OVCAR5 CBPR cells

Accession ID	Gene names	Protein names^a^	Fold change (CBPR/Parental)	p value
P32455	GBP1	Interferon-induced guanylate-binding protein 1	3.92	0.0011
P11413	G6PD	Glucose-6-phosphate 1-dehydrogenase	**3.64**	**0.0002**
P17301	ITGA2	Integrin alpha-2	**3.61**	**0.0001**
Q16555	DPYSL2	Dihydropyrimidinase-related protein 2	3.58	0.0005
Q12913	PTPRJ	Receptor-type tyrosine-protein phosphatase eta	3.24	0.0208
Q15582	TGFBI	Transforming growth factor-beta-induced protein ig-h3	**3.13**	**0.0135**
Q15738	NSDHL	Sterol-4-alpha-carboxylate 3-dehydrogenase, decarboxylating	3.05	0.0005
P15121	AKR1B1	Aldose reductase	**3.00**	**0.0017**
P51572	BCAP31	B-cell receptor-associated protein 31	2.89	< 0.0001
Q13642	FHL1	Four and a half LIM domains protein 1	2.59	0.0227
P06756	ITGAV	Integrin alpha-V;Integrin alpha-V heavy chain;Integrin alpha-V light chain	**2.52**	**0.0014**
P00492	HPRT1	Hypoxanthine–guanine phosphoribosyltransferase	2.48	0.0008
Q13636	RAB31	Ras-related protein Rab-31	2.45	0.0146
Q13501	SQSTM1	Sequestosome-1	**2.12**	**0.0293**
P56199	ITGA1	Integrin alpha-1	**2.10**	**0.0478**
O94808	GFPT2	Glutamine–fructose-6-phosphate aminotransferase [isomerizing] 2	**2.07**	**0.0086**
P07099	EPHX1	Epoxide hydrolase 1	2.02	0.0216
P21333	FLNA	Filamin-A	**2.01**	**0.0028**

^a^Proteins in bold are validated at the mRNA and/or protein levels

**Table 2:** Proteins downregulated in OVCAR5 CBPR cells

Accession ID	Gene names	Protein names	Fold change (CBPR/Parental)	p value
O95810	SDPR	Serum deprivation-response protein	7.10	0.0172
P22676	CALB2	Calretinin	5.76	0.0033
Q16822	PCK2	Phosphoenolpyruvate carboxykinase [GTP], mitochondrial	4.78	0.0403
Q13740	ALCAM	CD166 antigen	3.68	0.0216
Q86UP2	KTN1	Kinectin	3.54	0.0399
Q14141	SEPT6	Septin-6	3.44	0.0393
Q16658	FSCN1	Fascin	2.66	0.0052
Q13085	ACACA	Acetyl-CoA carboxylase 1; Biotin carboxylase	2.34	0.0003
Q9NP81	SARS2	Serine–tRNA ligase, mitochondrial	2.32	0.0059
Q9Y570	PPME1	Protein phosphatase methylesterase 1	2.30	0.0118
Q14315	FLNC	Filamin-C	2.25	0.0192
P13726	F3	Tissue factor	2.20	0.0359
Q15084	PDIA6	Protein disulfide-isomerase A6	2.13	0.047
P24666	ACP1	Low molecular weight phosphotyrosine protein phosphatase	2.11	0.0112

### Investigation of carboplatin sensitivity in OVCAR5 parental and OVCAR5 CBPR cells

Following multiple cycles of carboplatin treatment, cytotoxicity assays confirmed a fourfold increase in IC_50_ of carboplatin in OVCAR5 CBPR cells compared to OVCAR5 parental cells (Additional file 1: Fig. S1). Consistent with findings in the cytotoxicity assays, mRNA expression of drug resistance genes, *ABCG2* (2.24-fold; *p* < 0.01), was also upregulated in OVCAR5 CBPR cells compared to their carboplatin-sensitive counterparts (Additional file 1: Fig. S1).

### Proteomics analysis revealed differential expression of EMT modulators in carboplatin resistant cells

Among the upregulated proteins, interferon-induced guanylate-binding protein 1 (GBP1) topped the list. This was followed by glucose-6-phosphate dehydrogenase (G6PD), a protein crucial for the pentose phosphate pathway (PPP), followed by several proteins essential for ECM remodeling such as integrin alpha-2 (ITGA2), integrin alpha-V (ITGAV), integrin alpha-1 (ITGA1), filamin-A (FLNA1) and transforming growth factor induced protein [TGFBI, also known as βig-H3 (Table 1)]. Proteins involved in the synthesis of polyol aldoreductase (AKR1B1), glycan and protein glycosylation through hexosamine biosynthetic pathway glutamine-fructose-6-phosphate aminotransferase (GFPT2) and essential protein in the purine salvage pathway, hypoxanthine–guanine phosphoribosyltransferase (HPRT1) were also upregulated in OVCAR5 CBPR compared to OVCAR5 parental cells (Table 1). Among the downregulated proteins, serum deprivation-response protein (SDPR), calretinin (CALB2), CD166 antigen (ALCAM), kinectin (KTN1), septin-6 (SEPT6), fascin (FSCN1) and filamin-C (FLNC1) were in the list (Table 2). The downregulated proteins also included metabolism-associated proteins such as phosphoenopyruvate carboxykinase 2 (PCK2); mitochondrial cetyl-CoA carboxylase 1 (ACACA); biotin carboxylase.

Of the 18 proteins with an upregulated expression in OVCAR5 CBPR cells, text mining with PubMed identified 8 proteins that have been associated with EMT previously. A list of these proteins and their involvement in EMT are summarized in Table 3. Enrichment analyses using the STRING software (version 11) [24] also revealed an overrepresentation of GO (gene ontology) biological processes consistent with key events in EMT (in bold in Table 4). No pathway was enriched amongst the downregulated proteins.

**Table 3:** EMT-associated proteins with enhanced expression in OVCAR5 CBPR cells

Accession ID	Gene names	Protein names^a^	Fold change (CBPR/parental)	*p* value	Involvement in EMT
P17301	ITGA2	Integrin alpha-2	3.61	0.0001	- Ectopic expression of ITGA2 induced VIM and CDH2 expression while downregulated CDH1 [23]
Q15582	TGFBI	Transforming growth factor-beta-induced protein ig-h3	3.13	0.0135	- Enhanced expression of TGFBI stimulates invasive progression in vitro and correlates with poor prognoses in cancer patients [25, 29]
P15121	AKR1B1	Aldose reductase	3.00	0.0017	- High expression of correlates with aggressive and invasion tumor phenotype [74]
P06756	ITGAV	Integrin alpha-V; Integrin alpha-V heavy chain; Integrin alpha-V light chain	2.52	0.0014	- Downregulation of ITGAV via the anti-tumor miR-9-3p inactivates EMT, suppresses proliferation and metastasis in vitro [11] - ITGAV interference inhibited tumor growth through abolishing TGFβ1-SMAD signaling [96]
Q13501	SQSTM1	Sequestosome-1	2.12	0.0293	- Ectopic expression of SQSTM1 led to morphological and molecular changes in vitro, including spindle-shaped cells, low CDH1 expression, high levels of CDH2, VIM and SNAI1 [97]
P56199	ITGA1	Integrin alpha-1	2.10	0.0478	- ITGA1-collagen binding induced cell spreading and shift towards a mesenchymal morphology [71]
O94808	GFPT2	Glutamine–fructose-6-phosphate aminotransferase [isomerizing] 2	2.07	0.0086	- Immediate-early gene product induced by NF-κB in mesenchymal cells - *GFPT2* silencing reduced migration and invasion in ovarian cancer cells, HEY and SKOV3 [77]
P21333	FLNA	Filamin-A	2.01	0.0028	- Interacts with SMAD2 to promote EMT via SMAD2 target genes, *Snai1* and *MMP9* [98]

^a^Proteomics analysis identified 18 proteins with significantly higher expression in the OVCAR5 CBPR cells compared to that in OVCAR5 parental cells. 8 of these proteins have been reported to modulate EMT process. Fold changes are calculated using label-free quantification intensities of proteins from cell lysates (n = 3/group)

The role in EMT of these proteins are listed

**Table 4:** Over-representation of GO biological processes amongst upregulated proteins in OVCAR5 CBPR cells

GO term	Biological processes (GO)^a^	Count in gene set	False Discovery Rate
GO: 0030155	Regulation of cell adhesion	**6 of 623**	**0.0049**
GO:0065008	Regulation of biological quality	11 of 2559	0.0133
GO: 0051128	Regulation of cellular component organization	9 of 2306	0.0133
GO: 0033627	Cell adhesion mediated by integrin	**2 of 17**	**0.0133**
GO: 0032879	Regulation of localization	**9 of 2524**	**0.0133**
GO: 0030198	Extracellular matrix organization	**4 of 296**	**0.0133**
GO: 0007160	Cell–matrix adhesion	**3 of 1194**	**0.0138**
GO: 0009653	Anatomical structure morphogenesis	8 of 1992	0.0144
GO: 0000902	Cell morphogenesis	**5 of 626**	**0.0144**
GO: 0030154	Cell differentiation	**10 of 3457**	**0.0149**
GO: 0045785	Positive regulation of cell adhesion	**4 of 375**	**0.0151**
GO: 0040011	Locomotion	**6 of 1144**	**0.0163**
GO: 0007166	Cell surface receptor signaling pathway	8 of 2198	0.0163
GO: 0010810	Regulation of cell-substrate adhesion	**3 of 189**	**0.0182**
GO: 1900024	Regulation of substrate adhesion-dependent cell spreading	**2 of 47**	**0.0197**
GO: 0000904	Cell morphogenesis involved in differentiation	**4 of 498**	**0.0197**
GO: 0010769	Regulation of cell morphogenesis involved in differentiation	**3 of 263**	**0.0275**
GO: 0030334	Regulation of cell migration	**4 of 753**	**0.0492**

^a^GO enrichment analysis using the *Homo sapiens* gene database was performed on genes which encode the 18 upregulated proteins identified in the OVCAR5 CBPR cells. GO identifier, description of the biological processes and false discovery rate are listed. “Count in gene” set refers to the number of genes in our dataset that are mapped to the total number of genes in the respective category

### Validation of candidate proteins at mRNA and protein levels

**Fig. 1:**
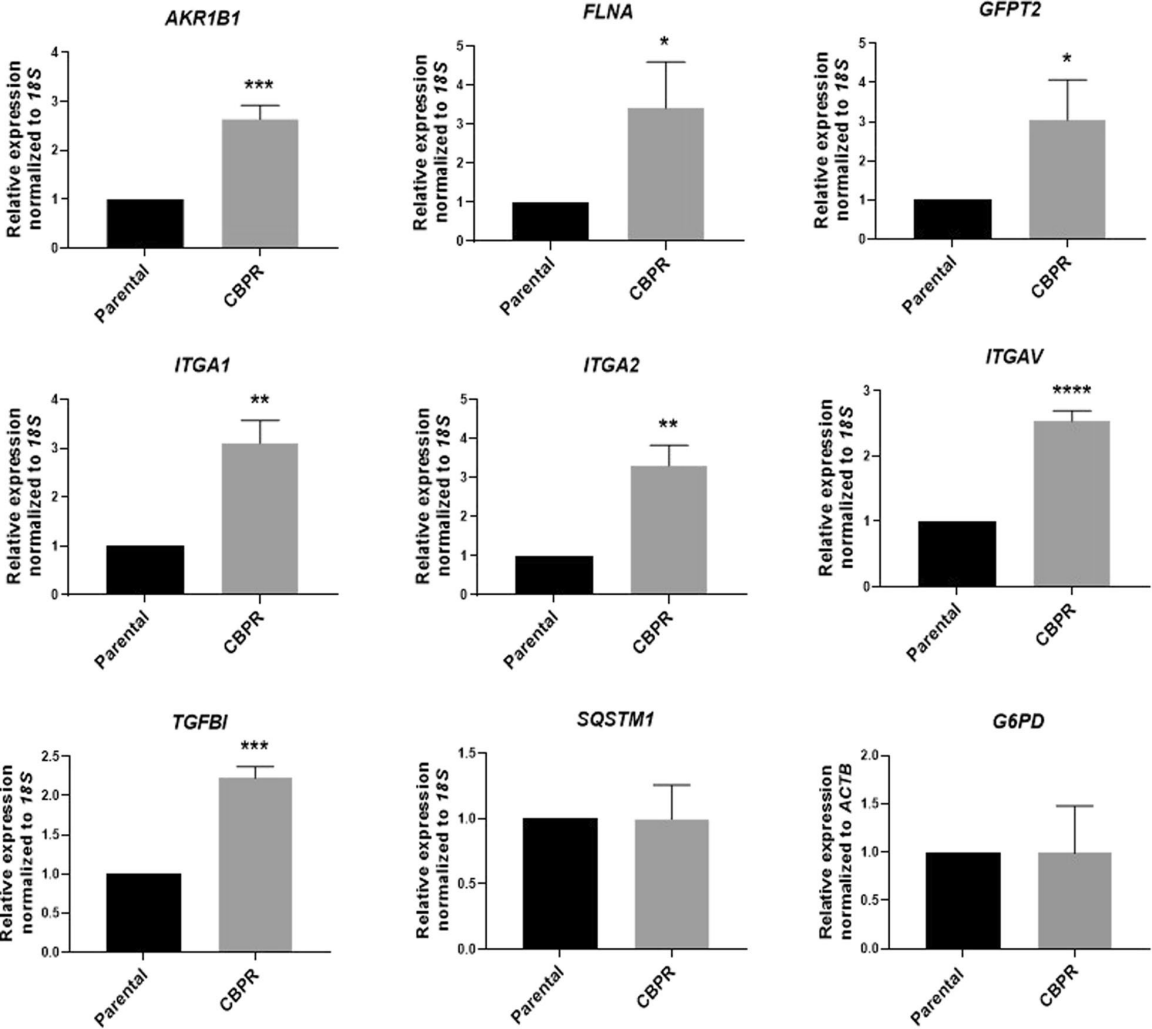
mRNA expression of EMT modulators in OVCAR5 cells. mRNA expression of target genes was evaluated in OVCAR5 CBPR cells compared to OVCAR5 parental cells as described in the Methods. mRNA expression was normalized to that of 18S or ACTB. n = 3; mean ± SD; student’s *t* test; *p < 0.05, **p < 0.01, ***p < 0.001, ****p < 0.0001 when compared to OVCAR5 parental cells

**Fig. 2:**
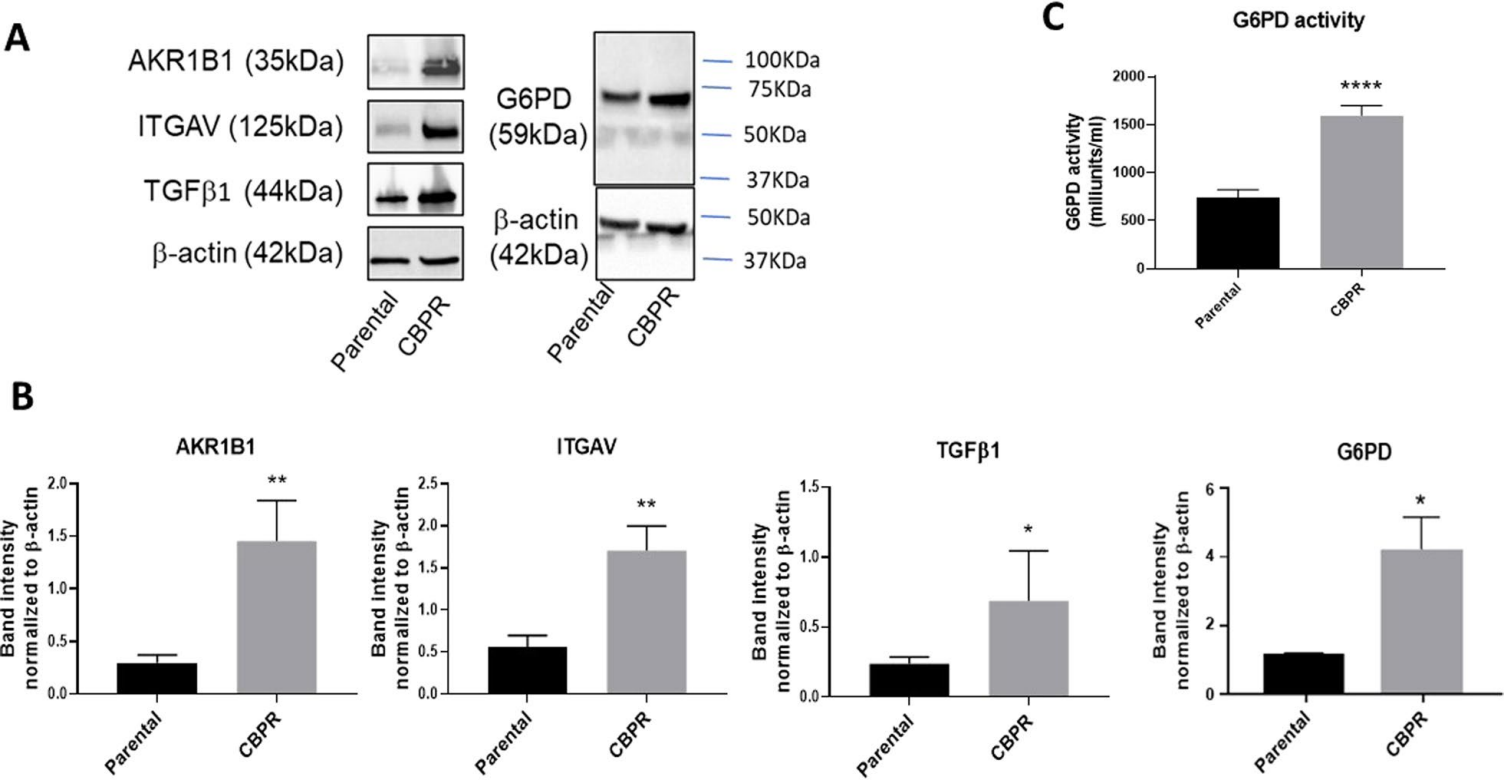
Protein expression of EMT modulators and activity of G6PD in OVCAR5 cells. **A** Upregulated of AKR1B1 (35 kDa), ITGAV (125 kDa), TGFβ1 (44 kDa) and G6PD (59 kDa) protein expression was confirmed using western blot. **B** Densitometry analyses of AKR1B1, ITGAV, TGFβ1 and G6PD were normalized against loading control β-actin (42 kDa). n = 3; mean ± SD; student’s t test; *p < 0.05, **p < 0.01 when compared to OVCAR5 parental cells. **C** G6PD activity was measured using a colorimetric based assay (MAK015, Sigma Aldrich) according to manufacturer’s instruction. n = 3; mean ± SD; student’s *t* test; ****p < 0.0001 when compared to OVCAR5 parental cells

We validated the mRNA expression of some of the candidate proteins by quantitative real-time PCR. Significantly upregulated mRNA expression of *AKR1B1* (2.64-fold; *p* < 0.001), *FLNA* (3.41-fold; *p* < 0.05), *GFPT2* (2.18-fold; *p* < 0.05), *ITGA1* (2.84-fold; *p* < 0.05), *ITGA2* (3.47-fold; *p* < 0.05), *ITGAV* (2.54-fold; *p* < 0.0001) and *TGFBI* (2.23-fold; *p* < 0.001) was confirmed in OVCAR5 CBPR cells compared to the parental OVCAR5 cells (Fig. 1). In contrast, mRNA levels of *SQSTM1* and *G6PD* showed no significant difference between the carboplatin sensitive OVCAR5 cells and their resistant counterparts (Fig. 1). Given their involvement in EMT cascade, we further analyzed the expression of G6PD, AKR1B1 and ITGAV by western blot. We also examined the protein expression of TGFβ (gene TGFB1), a protein that induces *TGFBI* and is implicated in EMT in ovarian cancer [26]. Not only did all three targets show significant induction at the mRNA levels, but their encoded proteins were also significantly upregulated in OVCAR5 CBPR cells (Fig. 2A, B). Despite a lack of difference at the mRNA level, we showed that protein expression of the enzyme G6PD was significantly elevated in the resistant cells (Fig. 2A, B). We also demonstrate that OVCAR5 CBPR cell line has significantly greater G6PD activity compared to parental OVCAR5 cell line (Fig. 2C), suggesting potential involvement of this protein in the biology of OVCAR5 CBPR cells.

### Expression of classical EMT markers in OVCAR5 cells

**Fig. 3:**
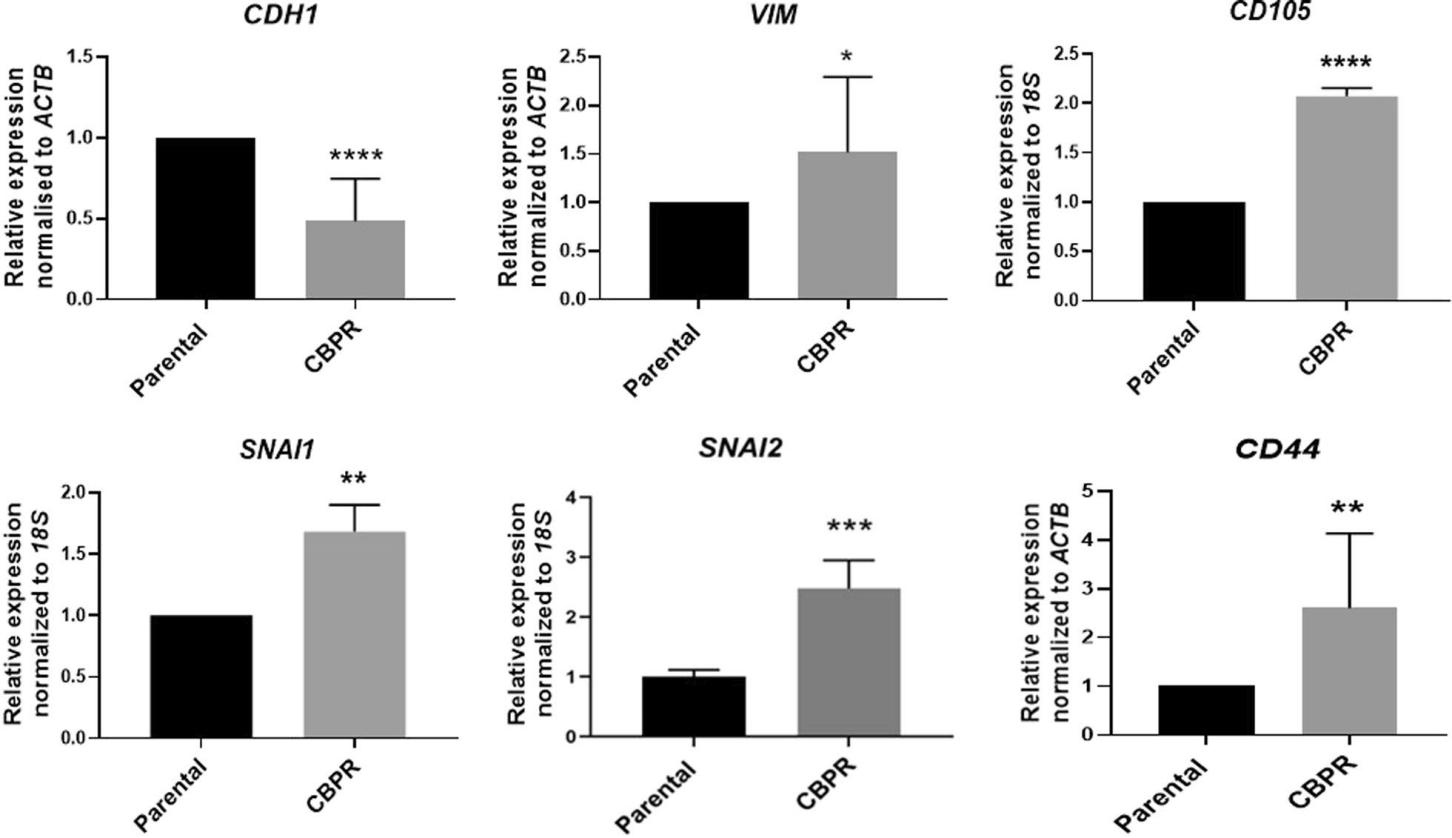
Evaluation of the expression of EMT markers in OVCAR5 CBPR versus parental OVCAR5 cells. mRNA expression of EMT markers, *CDH1***,** *VIM*, *SNAI1*, SNAI2, CD44 and **(***CD105* in OVCAR5 CBPR cells compared to OVCAR5 parental cells as described in Methods. mRNA expression was normalized to *ACTB* or *18S.* n = 3; mean ± SD; student’s *t* test; *p < 0.05, **p < 0.01, ***p < 0.001, ****p < 0.0001 when compared to OVCAR5 parental cells

The upregulation of EMT modulators in OVCAR5 CBPR cells prompted our investigation of the expression of classical EMT markers in these cells. Using quantitative real-time PCR, we identified a significant upregulation of mesenchymal markers, vimentin (*VIM*; 1.54-fold; *p* < 0.05), snail 1 (*SNAI1*; 1.68-fold; *p* < 0.01), snail2 (*SNAI2*; 2.2-fold; p < 0.001), CD44 (2.5-fold; p < 0.01) and endoglin (*CD105*; 2.08-fold; *p* < 0.0001) (Fig. 3). On the contrary, expression of epithelial marker, E-cadherin (*CDH1*; -1.59-fold; *p* < 0.0001) was significantly downregulated in the carboplatin-resistant cells (Fig. 3).

**Fig. 4:**
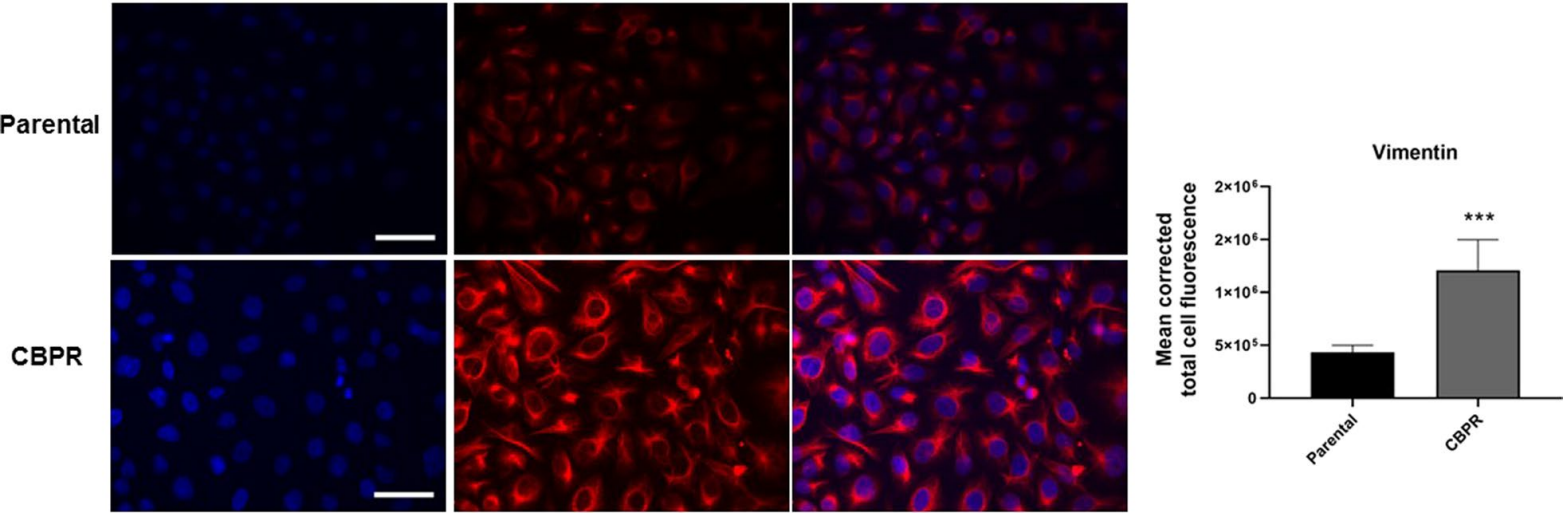
Vimentin expression in OVCAR parental and OVCAR5 CBPR cells. Representative images of immunofluorescence staining for nuclei (DAPI; blue) and vimentin (Alexa Fluor 494; red) at 40× magnification; scale bar = 100 µm. Quantification of vimentin fluorescent intensities was conducted using Image J software. n = 8 images, mean ± SD; Mann Whitney test; ****p < 0.001 when compared to OVCAR5 parental cells

We also performed immunofluorescence for the mesenchymal marker, vimentin, on these cells. A significantly stronger immunofluorescence staining for vimentin was observed in OVCAR5 CBPR cells compared to their parental cells (p < 0.001; Fig. 4), consistent with its significantly elevated mRNA expression and reduced expression of E-cadherin (Fig. 3).

### OVCAR5 CBPR cells exhibited higher migratory capacity with lower proliferation rate compared to parental OVCAR5 cells

**Fig. 5:**
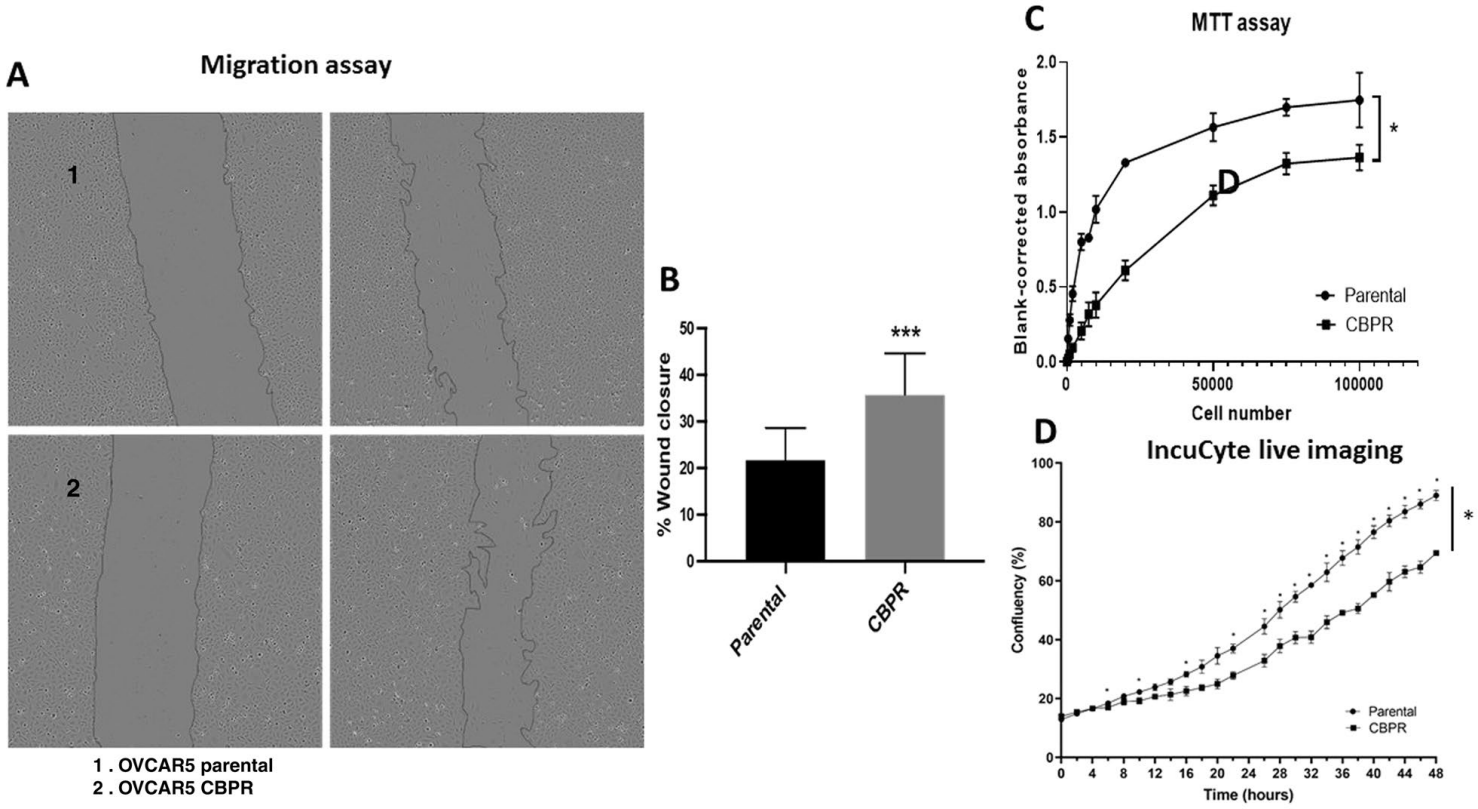
Migratory and proliferative abilities of OVCAR parental and OVCAR5 CBPR cells. **A**, **B** The migratory capacity of OVCAR5 parental (1) and OVCAR5 CBPR (2) cells was investigated using a wound healing assay. Representative images of wounds at 0 and 9-h time points. Percentage of wound closure was quantified using Image J software. n = 3; mean ± SD; student’s *t* test; ***p < 0.01 when compared to OVCAR5 parental cells. **C** Proliferation in OVCAR5 parental and CBPR cell lines was evaluated by MTT assay over a 24-h period. Wilcoxon matched pair signed rank test; n = 3; *p < 0.05 when compared to OVCAR5 parental cells. **D** Proliferation in OVCAR5 parental and CBPR cell lines was evaluated by Incucyte live imaging over a 48-h period. 2-way ANOVA with Šídák's multiple comparisons test; n = 5; *p < 0.05 when compared to OVCAR5 parental cells

To assess the functional impact of the altered EMT molecular profile in the OVCAR5 cells, we investigated cell motility using a scratch assay. Compared to the OVCAR5 parental cells, the carboplatin-resistant cells exhibited a significantly greater capacity to migrate, resulting in a higher percentage of wound closure (*p* < 0.01; Fig. 5A and B). Additionally, we also demonstrate that the difference in wound closure was not affected by proliferation. Data from our 24-h proliferation assays (MTT and IncuCyte^®^live imaging) revealed that OVCAR5 CBPR cells are less proliferative compared to their chemo-sensitive parental counterparts (Fig. 5C and D).

### Carboplatin-resistant cells showed lower glycolytic activities compared to its sensitive counterpart

**Fig. 6:**
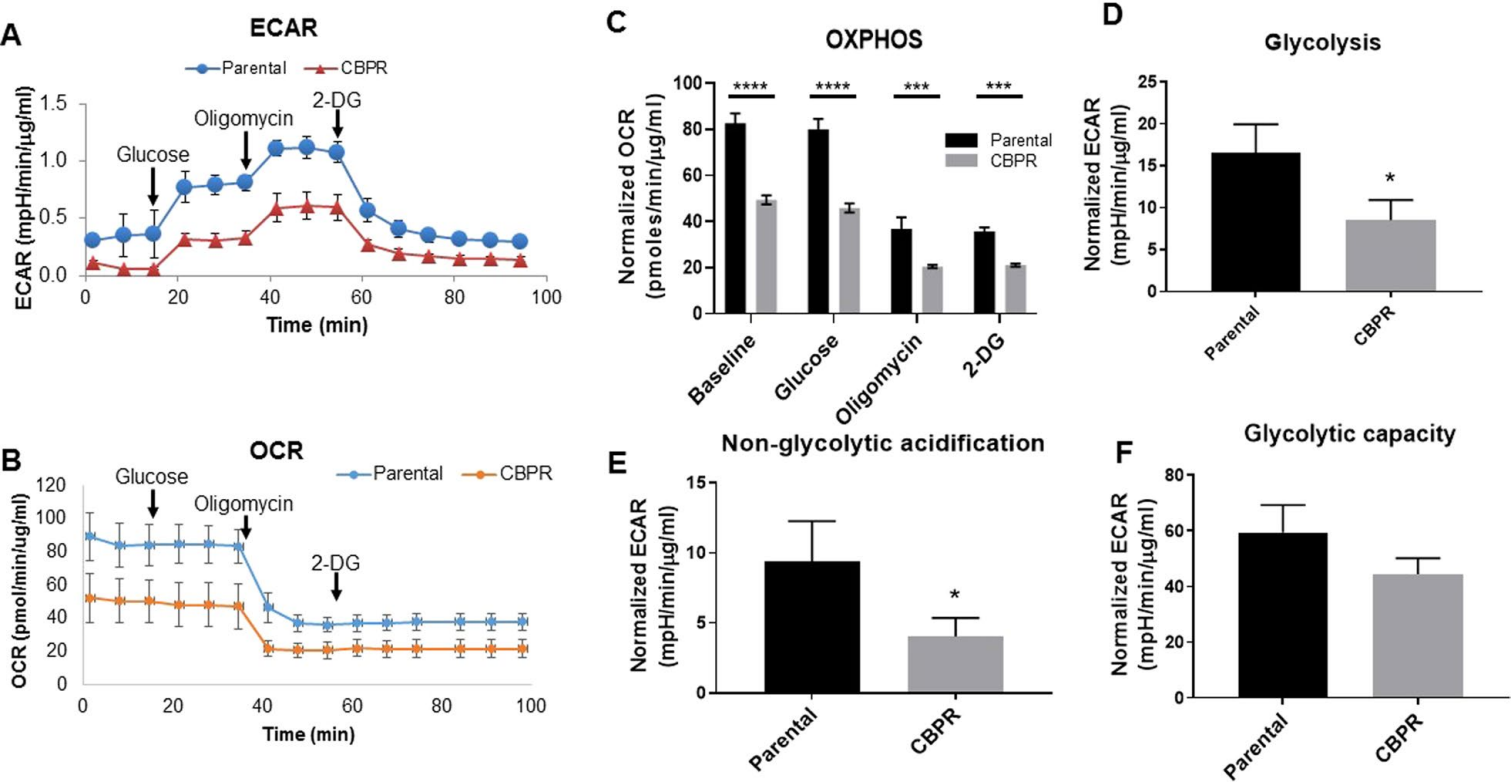
Cellular bioenergetics in OVCAR5 parental and OVCAR5 CBPR cells. Representative graph of measurements of (**A**) extracellular acidification rate (ECAR) following the sequential addition of glucose, oligomycin, an ATPase inhibitor and 2-deoxyglucose (2-DG), a competitive inhibitor for glucose of hexokinase, were used to calculate rate of **B** oxygen consumption rate (OCR), **C** oxidative phosphorylation (OXPHOS), **D** glycolysis, **E** non-glycolytic acidification and **F** glycolytic capacity. ECAR was normalized to total protein concentration. n = 3; mean ± SD; student’s *t* test; ****p < 0.0001, ***p < 0.001, *p < 0.05 when compared to OVCAR5 parental cells

Cancer cells are often reported to undergo metabolic reprogramming concurrently to promote EMT. From our proteomics data we also observed the upregulation of G6PD in OVCAR5 CBPR cells (3.64-fold; *p* < 0.0002; Table 1), the first enzyme in the pentose phosphate pathway, suggesting a shift in carbon flow from glycolytic flux to pentose phosphate pathway. This is consistent with enhanced mRNA and protein expression of this protein as shown in Figs. 1 and 2. Further to that, we also show 3.5-fold upregulation of G6PD activity in OVCAR5 CBPR cells compared to their control counterpart (Fig. 2C). To understand how OVCAR5 CBPR cells consume energy we investigated the cellular bioenergetics profile of parental and carboplatin-resistant ovarian cancer cells using an extracellular flux assay which measures the oxygen consumption rate (OCR) to determine mitochondrial oxidative phosphorylation (OXPHOS) and extracellular acidification rate (ECAR), a measure of glycolysis. Compared to the carboplatin sensitive OVCAR5 parental cells, the resistant counterpart displayed a reduced extracellular acidification rate (ECAR), which is indicative of low glycolysis, as seen in the representative graph (Fig. 6A). A reduction in oxygen consumption rate suggesting a lower rate of mitochondrial respiration (or OXPHOS) was seen in the OVCAR5 CBPR cells at baseline as well as upon 2-deoxy glucose (2-DG) injection to shut down glycolysis and drive OXPHOS (Fig. 6B and C). A significantly lower extracellular acidification rate (ECAR), which measures glycolysis, was also observed (*p* < 0.05; Fig. 6D). The non-glycolytic acidification, a source of acidification other than glycolysis such as the TCA cycle and/or glycogenolysis (breakdown of glycogen to glucose) [27], also showed a significant decline in the chemoresistant cells (*p* < 0.05; Fig. 6E). In response to the shutdown of OXPHOS by oligomycin, a compound that inhibits ATP synthase, the cells were forced to rely on glycolysis to meet energy demands. Our data revealed that, upon treatment of oligomycin, sensitivity to chemotherapeutic agents did not alter the maximum capacity of these cells to generate ATP through glycolysis (Fig. 6F).

### Ovarian cancer patients with platinum-resistant disease showed increased expression of EMT modulators

**Fig. 7:**
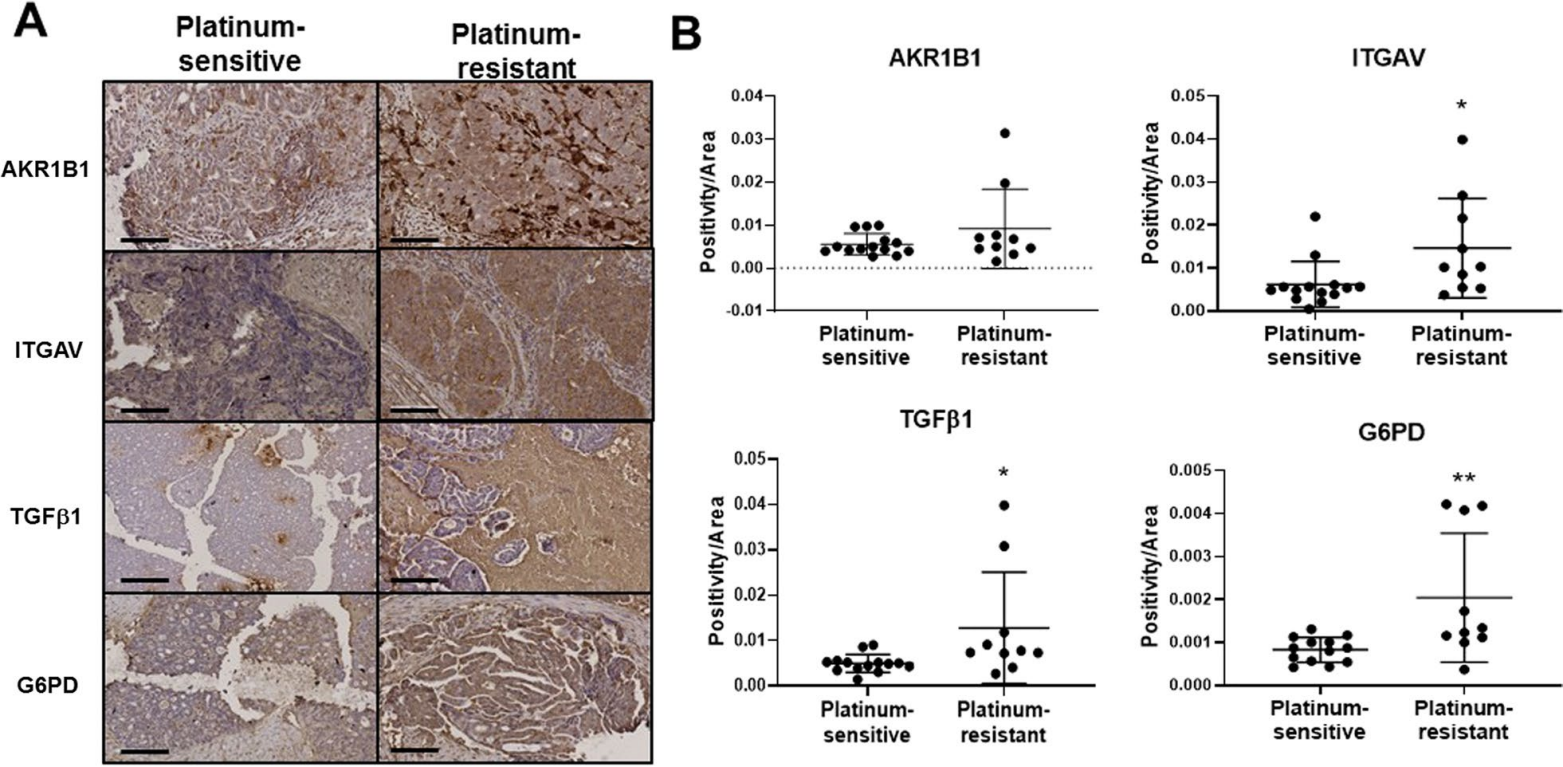
Immunohistochemical staining for AKR1B1, ITGAV, TGFβ1 and G6PD in ovarian tumor samples collected from patients with platinum sensitive and resistant disease. Immunohistochemistry for AKR1B1, ITGAV, TGFβ1 and G6PD was performed on 15 platinum-sensitive and 10 platinum-resistant tumor tissues collected from ovarian cancer patients. **A** Representative images of staining on platinum-sensitive and platinum-resistant tumor tissues collected from ovarian cancer patients were displayed at 20 × magnification; scale bar = 100 μm. Quantification of DAB staining for **B** AKR1B1, ITGAV, TGFβ1 and G6PD was performed using Aperio ImageScope software; mean ± SD; Mann–Whitney test; outliers greater than 2 SD are excluded where n = 13–14 in platinum-sensitive group. **B** *p < 0.05, **p < 0.01 when compared to platinum-sensitive group

**Fig. 8:**
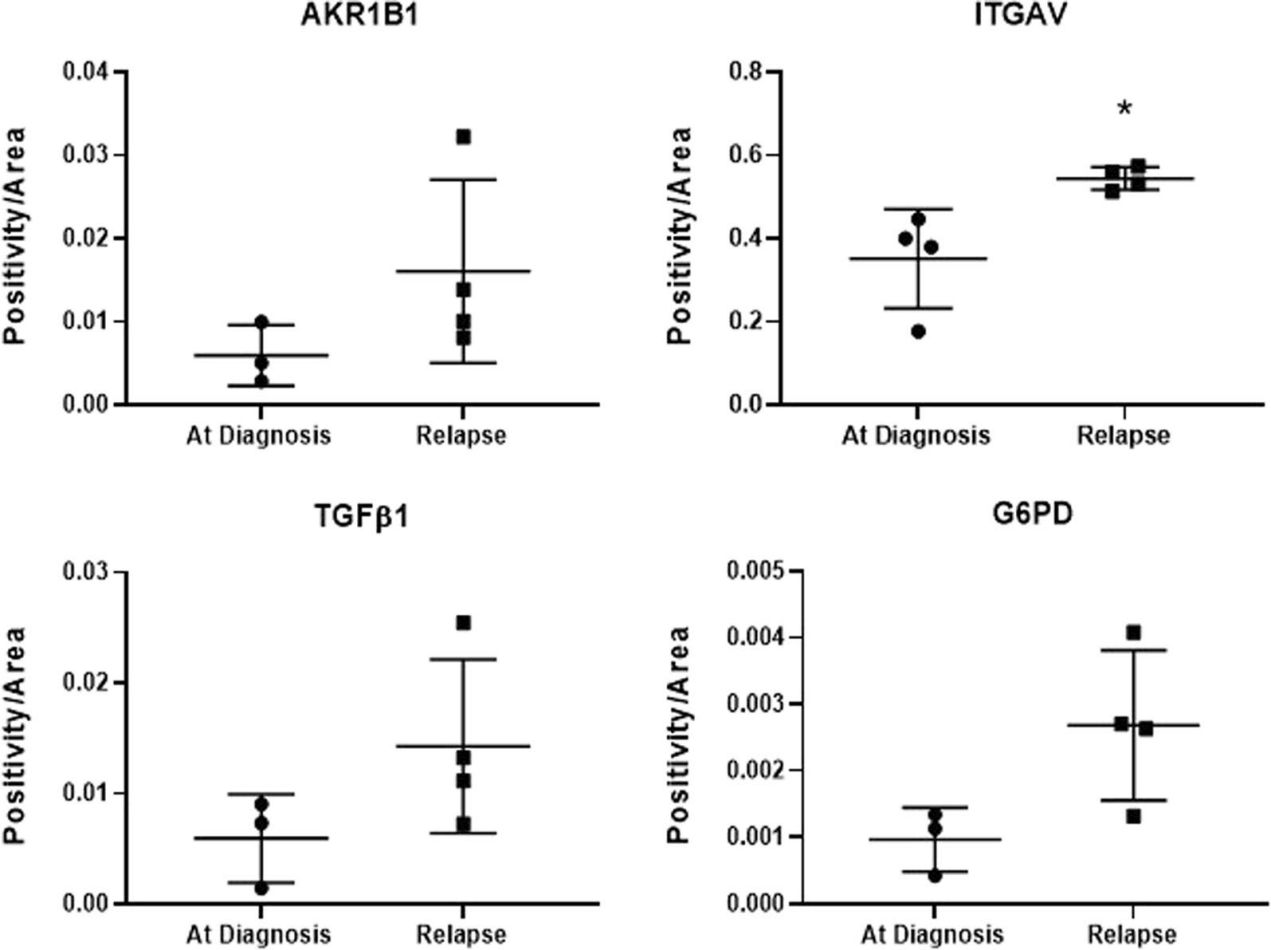
Immunohistochemical staining for AKR1B1, ITGAV, TGFβ1 and G6PD in matching ovarian tumor samples at diagnosis and at relapse. Immunohistochemistry on these samples was performed as described in Methods. Quantification of DAB staining for AKR1B1, ITGAV, TGFβ1 and G6PD was performed using Aperio ImageScope software. Mean ± SD; paired student’s *t* test; outliers greater than 2 SD are excluded where n = 3 in ‘at diagnosis’ group; *p < 0.05 in relapsed group when compared to initial diagnosis

We then assessed the expression of the identified EMT and metabolic modulators on platinum-resistant versus -sensitive tumors collected at diagnosis from ovarian cancer patients. Patients who responded to platinum drug (carboplatin) after six months of treatment with carboplatin were rendered platinum sensitive, while patients who developed resistance to carboplatin within six months of treatment were considered platinum resistant. Tumors from patients who are resistant to platinum showed stronger expression for AKR1B1, ITGAV, TGFβ1 and G6PD compared to those who are platinum sensitive (Fig. 7A and B). Amongst the matched initial diagnosed and relapsed samples (n = 4), a trend of increased expression of the EMT modulators and G6PD was observed but only ITGAV showed a significantly higher expression in relapse tumors compared to initial diagnosis (Fig. 8). Staining intensity has no association with age or progression-free survival (data not shown).

### Kaplan–Meier plot indicated EMT modulators are negatively associated with progression-free survival

**Fig. 9:**
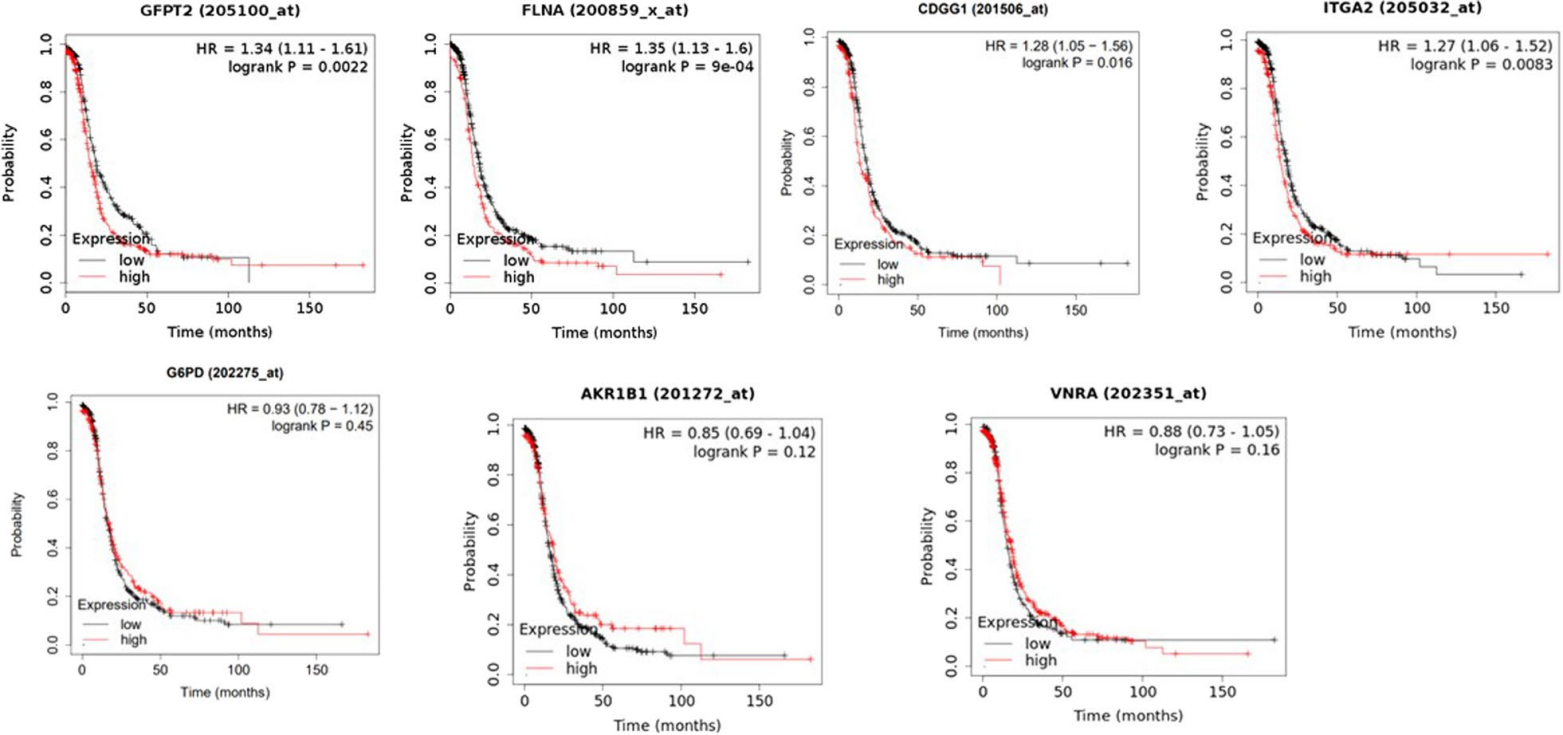
Kaplan–Meier plot of EMT modulators. The EMT modulators, *GFPT2*, *FLNA*, *CDGG1* (*TGFB1*), *G6PD, AKR1B1, VNRA* (*ITGAV*) and *ITGA2* were explored in a public gene expression dataset with a cohort of 738 patients with advanced stage (stages 3 and 4) high-grade serous ovarian cancers. All patients received chemotherapy treatment, all variants of TP53 (wild type or mutant) were included. Biased arrays were excluded from analyses

Expression of selected genes and their association with progression-free survival were explored in gene expression TCGA dataset of 738 patients with advanced-stage (stages 3 and 4) high-grade serous ovarian cancer. High expression of several EMT modulators identified by proteomics including GFPT2, FLNA, ITGA2and TGFBI (CDGG1) were negatively associated with progression-free survival (Fig. 9). However, no association between G6PD, AKR1B1 and VNRA (ITGAV) and progression-free survival was observed (Fig. 9). ITGA1 was not included in the datasets for our analysis.

### Analysis based on TIMER database

**Fig. 10:**
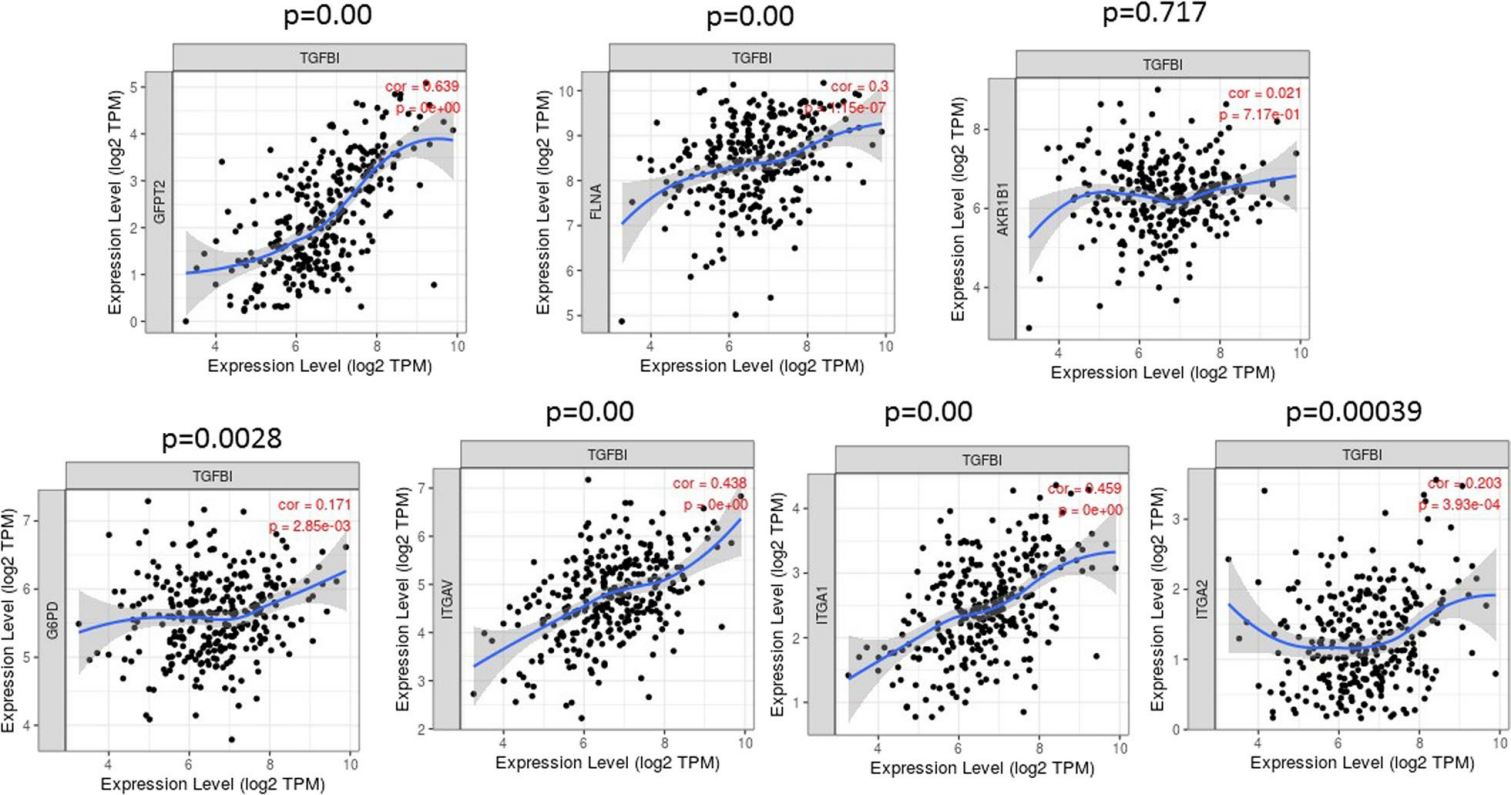
Correlation of TGFBI expression with proteomics identified and validated proteins in OVCAR5 CBPR cell line by TIMER database. The correlative expression of TGFBI with GFPT2, FLNA, AKR1B1, G6PD, ITGAV, ITGA1 and ITGA2 was deduced by using ‘Gene Go’ module. The expression scatterplots between TGFBI and other significantly upregulated protein in OVCAR5CBPR cell lines (GFPT2, FLNA, AKR1B1, G6PD, ITGAV, ITGA1 and ITGA2) in a cohort of 303 samples of serous ovarian cystadenocarcinomas are presented. Statistical significance was deduced using Spearman’s rho value and adjusted for tumor purity

“Gene module’ and ‘Diff Exp module’ within TIMER database were used to assess to analyze the correlative expression of TGFBI with different EMT modulators identified by proteomics on 303 serous ovarian cystadenocarcinomas. TGFBI was chosen as it is among the top six upregulated proteins in CBPR OVCAR5 cell line and is a known regulator of EMT. Its expression was higher in tumors resistant or refractory to 1st-line chemotherapy compared with sensitive tumors [28]. The statistical significance of the scatterplots between TGFBI expression and different genes of interest in a cohort of 303 serous ovarian cystadenocarcinomas was deduced using Spearman’s rho value. Amongst the proteomics identified and validated genes, the expression of TGFBI significantly positively correlated with the expression of GFPT2, FLNA, G6PD, ITGAV, ITGA1 and ITGA2 within the serous ovarian cystadenocarcinomas in TIMER database (Fig. 10). However, no significant expression correlation was observed between TGFBI and AKR1B1.

**Fig. 11:**
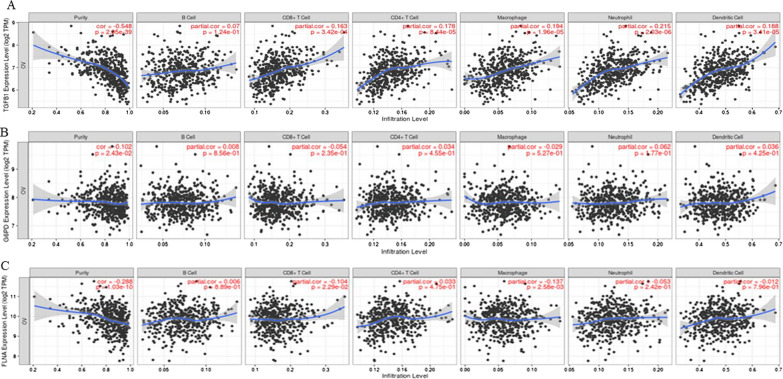
Correlation of TGFB1, G6PD, FLNA with infiltration of different subsets of immune cells analysed by TIMER database. The infiltration of B cells, CD8^+^ T cells, CD4^+^T cells, macrophages, neutrophils, and dendritic cells in relation to **A** TGFB1, **B** G6PD and **C** FLNA expression was deduced by using TIMER dataset. Statistical significance was evaluated using Spearman’s rho value. The gene expression levels against tumor purity are listed on the top of each scatter plot. Genes highly expressed in the microenvironment are expected to have negative associations with tumor purity, while the opposite is expected for genes highly expressed in the tumor cells

**Fig. 12:**
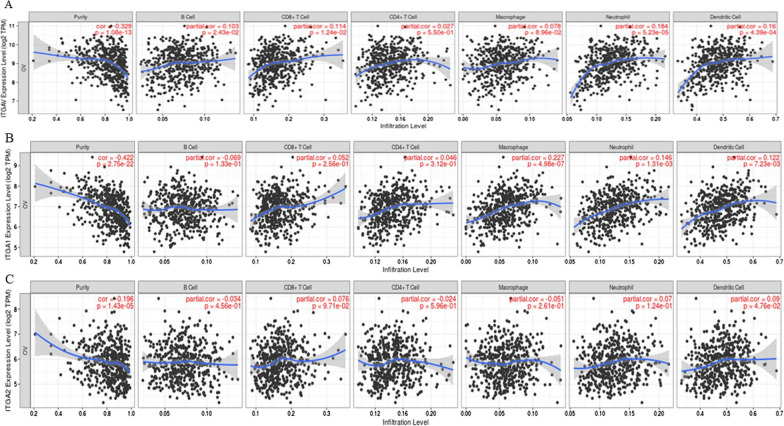
Correlation of ITGAV, ITGA1 and ITGA2 with infiltration of different subsets of immune cells analysed by TIMER database. The infiltration of B cells, CD8^+^ T cells, CD4^+^T cells, macrophages, neutrophils, and dendritic cells in relation to **A** ITGAV, **B** ITGA1 and **C** ITGA2 expression was deduced and evaluated as described in Fig. 11

In addition to differential correlation of genes, we used Gene module within the TIMER dataset, which allowed us to visualize the correlation of expression of genes of our interest with immune cell infiltration level in 303 serous ovarian cystadenocarcinomas. Spearman’s rho value was used to assess the significance between the gene of interest and different subtypes of immune cell infiltration. We selected TGFB1, G6PD, ITGAV, ITGA1, FLNA and ITGA2 genes and their association with different subtypes of immune cell infiltration was analyzed. These genes were selected as they showed a significant positive correlation by the Diff Exp module, and they play an active role in redox sustenance and ECM-remodeling consistent with carboplatin-induced EMT in OVCAR5 cells as discussed above. Our analysis showed a statistically significant positive infiltration of CD8^+^ T cells, CD4^+^ T cells, macrophages, neutrophils, and dendritic cells with TGFB1 expression (Fig. 11A, Table 5). The infiltration of B cells on the other hand, was not significant with TGFB1 expression. On the other hand, no association of G6PD with any of the immune cell subtype was noted (Fig. 11B, Table 5), while FLNA significantly correlated with the expression CD8 + T cells and macrophages (Fig. 11C, Table 5). ITGAV expression was significantly associated with positive infiltration of B cells, CD8^+^T cells, macrophage, neutrophils, and dendritic cells (Fig. 12A, Table 5). The expression of ITGA1 positively correlated with the expression of macrophages, neutrophils, and dendritic cells (Fig. 12B, Table 5), while ITGA2 showed only positive correlation with dendritic cells (Fig. 12C, Table 5). In summary, dendritic cells were positively regulated by all the genes, except FLNA, and CD4^+^T cells only correlated positively with TGFB1 expression analyzed in this study (Figs. 11 and 12, Table 5).

**Table 5:** Correlation between proteomic-identified proteins in CBPR OVCAR5 cell line and infiltration of immune cells deduced from Go Gene module of TIMER database

Proteomics identified protein	B cells partial correlation p value	CD8^+^T cells	CD4^+^ T cells	Macrophages	Neutrophils	Dendritic cells
TGFB1	0.054 p = 0.123	0.162 *p = 0.0003	0.178 *p = 0.00084	0.193 *p = 0.000019	0.214 *p = 2.03254E−06	0.187 *p = 0.000034
G6PD	0.008 p = 0.855	− 0.054 p = 0.235	0.034 p = 0.454	− 0.028 p = 0.526	0.061 p = 0.176	0.036 p = 0.425
FLNA	0.006 p = 0.889	0.186 *p = 0.000	0.0326 p = 0.474	− 0.137 *p = 0.002	− 0.053 p = 0.242	− 0.011 p = 0.795
ITGAV	0.102 *p = 0.102	− 0.103 *p = 0.022	0.027 p = 0.549	0.077 p = 0.089	0.183 *p = 0.000	0.159 *p = 0.000
ITGA1	− 0.068 p = 0.132	0.051 p = 0.256	0.046 p = 0.311	0.227 *p = 0.000	0.146 *p = 0.001	0.122 *p = 0.007
ITGA2	− 0.034 p = 0.456	0.075 p = 0.097	− 0.024 p = 0.595	− 0.051 p = 0.260	0.070 p = 0.124	0.090 *p = 0.047

* indicate the p values

## Discussion

Despite an initial good response to carboplatin, most ovarian cancer patients relapse due to an acquired or intrinsic resistance to platinum-based chemotherapy. In vitro studies have elucidated several cellular mechanisms driven by complex epigenetic and genetic changes to escape antitumor toxicities. Mechanisms of platinum resistance include, but not limited to, reduced accumulation of drugs due to impaired influx or potent efflux/secretion mechanisms, accumulation of reactive oxygen species (ROS) and simultaneous detoxification by antioxidants, elevated levels of DNA damage repair mechanisms, changes in membrane protein trafficking, aberrant protein and gene expressions altering drug transport and uptake, as well as pathways such as those hindering apoptosis and inducing EMT in response to chemotherapy treatment in surviving cells [29]. Platinum resistance has become the focus of cancer therapeutic development in recent years although no effective management has been shown to circumvent this phenomenon in resistant/refractory ovarian cancer patients. In this study, we sought to explore the proteome of ovarian cancer cell lines, the carboplatin sensitive OVCAR5 cells and their resistant counterparts, to identify proteins associated with carboplatin resistance and the associated functional consequences that may explain the resistance phenotype. We report an upregulation of several novel EMT modulators in the carboplatin-resistant cells including G6PD, AKR1B1, ITGAV, ITGA2, ITGA1, FLNA, GFPT2 and TGFBI. Functional analyses in vitro showed low proliferation and enhanced migratory features in the carboplatin-resistance cells, compared to parental chemo naïve cell line. An enhanced expression of EMT and metabolism regulators including G6PD and AKR1B1 and TGFβ1 observed in vitro in carboplatin resistant cell line was confirmed in platinum resistant and relapsed human ovarian tumor samples, compared to chemo naïve human tumors. In short, carboplatin-resistant cells acquired a plastic phenotype, and were less proliferative and more migratory. These slow-cycling resistant cells expressed drug resistance and CSC markers (ABCG2, CD44, CD105) and exhibited a low OXPHOS-glycolysis signature.

During cancer progression, tumor cells procure migratory feature imitating the phenotypic characteristics of EMT that occurs during embryogenesis and wound healing [30]. This feature of EMT is widely recognized as the central component of disseminated cancer cells that form distant metastasis [31]. Various mechanisms of enhanced migration in EMT-transformed cells have been elucidated, among those widely recognized is the loss of epithelial junction protein E-cadherin (CDH1) and replacement by other cadherins and intermediate filament protein vimentin (VIM) which facilitate mesenchymal transformation for single cell or collective migration [32]. In this study, we demonstrate decreased expression of E-cadherin and enhanced expression of vimentin, consistent with enhanced migration in CBPR OVCAR5 compared to their parental OVCAR5 cell line.

Our study also demonstrates reduced bioenergenetics in EMT transformed CBPR OVCAR5 cells, consistent with reduced proliferation in the resistant cell line compared to parental OVCAR5 cells. The link between EMT and reduced proliferation has been observed in many cellular model systems. Importantly, reduced proliferation in EMT-transformed disseminating cells at the invading margins of tumors has been noted. This presumably occurs to facilitate the re-establishment of migrating cells in their secondary niches to promote secondary growth and is enabled by the overexpression of cell cycle related cyclin-dependent kinase inhibitors p16(INK4a), p21(Cip1) or p27(Kip1) [33]. These observations are consistent with EMT programs described in cells under conditions that are not permissive for cellular growth, such as hypoxia and anoikis [34, 35]. It is also coherent with previous observations in chemotherapy surviving EMT transformed residual cells which had decreased bioenergetic demands, potentially to reduce their susceptibility to hostile cytotoxic microenvironment to enhance sustenance after therapeutic treatment [4]. Moreover, clinicopathological studies have demonstrated reduced proliferation in EMT-transformed circulating tumor cells (CTCs) and disseminated tumor cells during and after chemotherapy treatment [36]. In fact, slow-cycling phenotype in cancer cells has been recognized as a core mechanism for therapy resistance [37, 38]. Isolated populations of slow-growing, label-retaining colon cancer and breast cancer cells had enhanced survival when exposed to a third-generation platinum derivative, oxaliplatin, compared to non-labelled cells [38].

Altered expression of ECM components and integrins, have been widely documented in EMT-induced cancer cells preceding and through the metastatic process [39]. In ovarian cancer, the expression of several integrin complexes, including αvβ1, which binds fibronectin, and integrins α1β1 and α2β1, which interact with collagen, have been shown to contribute to cancer progression and chemoresistance [40]. Enhanced expression of ITGAV (αv subunit), ITGA1 (α1 subunit) and ITGA2 (α2 subunit) in OVCAR5 CBPR cells is indicative of ECM changes consistent with enhanced production of fibronectin and collagen observed in several malignancies and in most EMT-induced cancer models [41]. In addition, enhanced expression of filamin A (FLNA) may relate to cytoskeleton actin and tubulin reorganization as seen in different cancer models undergoing EMT [42].

In ovarian cancer, TGFβ1 induction has been shown to be a hallmark of EMT [43]. Cancer cells subjected to TGFβ1 treatment was found to become more motile and invasive [44]. TGFBI an ECM protein, with multiple functions in ovarian cancer is up regulated by TGFβ signaling pathway. Loss of TGFBI expression in HGSOC has been noted [19]. This occurs due to promoter hypermethylation of TGFBI promoter resulting in the silencing of the gene expression [19]. This promoter hypermethylation of TGFBI correlates with paclitaxel resistance in ovarian cancer [45]. In the same context, paclitaxel-resistant cells when treated with recombinant TGFBI protein showed increased paclitaxel sensitivity due to FAK-Rho dependent stabilization of microtubules [46]. These findings suggest that TGFBI plays an important role in chemoresistance. We thus investigated the expression of TGFβ1 and downstream targets following our observation of TGFBI overexpression in the mass spectrometry analysis of OVCAR5 CBPR cells. Enhanced expression of TGFβ1 protein as seen in our western blot analysis compliments the increased mRNA expression of TGFBI in OVCAR5 CBPR cells, suggesting an enhanced expression and functional activation of TGFβ1 in these cells. This is consistent with microarray studies that demonstrated significantly high expression of TGFBI in methotrexate, cisplatin, doxorubicin, vincristine, topotecan and paclitaxel-resistant ovarian cancer cell lines and reconciled those findings to changes in ECM remodeling in response to drug treatment [47].

Our proteomics findings and subsequent validation also demonstrated an enhanced expression of the ITGAV, ITGA1 and ITGA2 in OVCAR5 CBPR versus parental OVCAR5 cell lines. ITGAV expression has been shown to positively correlate with the molecular signature of mesenchymal cells and metastasis of cancer cells [7]. At the same token, TGFβ driven gemcitabine resistance and upregulated expression of ITGA1 has been noted in pancreatic ductal cell carcinoma, which promoted EMT and metastasis [48]. Similarly, overexpression of ITGA2 in esophageal squamous cell carcinoma cell lines promoted EMT and metastasis through FAK/AKT pathway [49] and also showed chemotherapy resistance in gastric cancer, while ITGA2 knockdown restored chemosensitivity by inducing apoptosis in chemoresistant cells [50]. These observations indicate a collective role of TGFBI, ITGAV, ITGA1 and ITGA2 in EMT-induced metastasis and chemotherapy resistance.

AKR1B1 also functions as a rate-limiting enzyme that catalyzes the reduction of glucose in the polyol pathway [51]. AKR1B1 overexpression has been shown to strongly correlate with the molecular profile of mesenchymal-like cells in various cancer models [52]. In lung cancer, high expression of AKR1B1 resulted in enhanced glutathione (GSH) synthesis and resistance to EGFR inhibitors in cell lines and xenograft models [51]. Whether the same aspect of AKR1B1 exists in OVCAR5 CBPR cells remains to be evaluated. We also report for the first-time an association of GFPT2 with chemotherapy resistant ovarian cancer. GFPT2 has been associated with enhanced glycosylation of proteins concomitant with mesenchymal functions and morphology and is regulated by GSH [53]. GFPT2 has been reported to initiate EMT in serous ovarian cancer by activating the hexosamine synthetic pathway to enhance the nuclear localization of β-catenin [54]. These observations suggest that both AKR1B1 and GFPT2 may work in conjunction to sustain the levels of GSH in CBPR OVCAR5 cells for their sustenance against the ROS insult triggered by platinum treatment.

Our proteomics data also revealed expression of G6PD to be higher in the OVCAR5 CBPR cells and this was consistent in platinum-resistant human ovarian tumors compared to their chemo-sensitive counterparts. This enzyme drives the first step of pentose phosphate pathway and converts glucose to ribose-5-phosphate required for nucleotide synthesis. This is consistent with the upregulation of hypoxanthine guanine phosphoribosyl transferase (HPRT1), an enzyme critical for the maintenance of purine salvage pathway. The upregulation of these rate-limiting enzymes could divert glucose metabolism through glycolysis towards pentose phosphate pathway and purine salvage pathways, which may explain the reduction of the glycolytic pathway in the OVCAR5 CBPR cells. Whilst not investigated in this study, pentose phosphate pathway also produces NADPH, which is central to the detoxification of ROS. The survival benefit of G6PD overexpression is exemplified in G6PD-deficient mice with high levels of oxidative damage in the brain [55]. Like other platinum antineoplastic drugs, carboplatin induces ROS production upon DNA binding. In resistant cancer cells, elevated expression of G6PD, AKR1B1 and GFPT2 counteracts the effects of ROS production as a prerequisite for cell survival and therapy resistance [56]. A recent study utilizing ovarian cancer patient-derived spheroids also reported significant association between cisplatin resistance, elevated levels of G6PD and enhanced level of GSH-producing enzymes in resistant cells [57]. These observations suggest the importance of chemotherapy-induced ECM remodeling (potentially through TGFBI) and ROS counteracting measures for the survival of chemotherapy stressed cancer cells.

Using multi-omic and bioenergetics analyses, a recent study demonstrated that high-OXPHOS ovarian cancer cells with higher ROS content exhibited greater sensitivity to taxane and platinum treatment compared to low-OXPHOS cells [58]. Given the pharmacological role of platinum compounds in initiating ROS production, tumor metabolic reprogramming to survive under therapy-induced oxidative stress is anticipated. This can be achieved by reducing intracellular ROS production through low levels of mitochondrial respiration, elevated dependency on glycolytic phenotype, or enhanced capacity of antioxidant detoxification [58, 59]. Our profiling of cellular bioenergetics using Seahorse extracellular flux assay revealed that the carboplatin-resistant cells have a low-OXPHOS low-glycolysis phenotype. The low metabolism and activated pathways responsible for ROS scavenging likely work in synergy to confer resistance to carboplatin-induced oxidative stress. The carboplatin resistant OVCAR5 cells also possess the ability to switch between glycolysis and OXPHOS, which indicates plasticity to adapt to metabolic stresses. In addition, they exhibited a lower rate of other non-glycolytic means of ATP generation such as the TCA cycle and/or glycogenolysis. The slower rate of cell growth but higher migratory capacity of the less metabolically active OVCAR5 CBPR cells may suggest that these cells undergo migration at the expense of proliferation. The downregulation of PCK2 (required for the conversion of oxaloacetate to pyruvate that feeds into the anabolic gluconeogenic pathway) and ACACA (a rate-limiting enzyme in de novo fatty acid synthesis that catalyzes the conversion of acetyl-CoA to malonyl-CoA) in OVCAR5 CBPR cells may also indicate a lack of dependency of these cells on anabolic gluconeogenic and fatty acid synthesis pathways. Further work needs to be done to investigate if these cells get metabolically ‘switched-on’ to support migration and reduced proliferation as reported in other slow-cycling cells.

The expression levels of carboplatin resistance-associated proteins, AKR1B1, ITGAV, TGFβ1 and G6PD, in platinum resistant and relapsed human ovarian cancer samples are consistent with our observations in vitro data on control and carboplatin-resistant cells. However, no prognostic role of AKRIBI, ITGAV and G6PD was evident in TCGA data and GSE (from Gene Expression Omnibus) datasets which included 738 high grade ovarian cancer patients that had undergone chemotherapy treatments. High expression of TGFBI, GFPT2, FLNA and ITGA2 on the other hand, were bad prognostic indicators in this patient cohort. Interestingly, the positive expression profiling of TGFBI with important ECM remodeling proteins such as GFPT2, FLNA, G6PD, ITGAV, ITGA1 and ITGA2 shown by TIMER datasets suggest a potential role of these proteins in remodeling ECM in response to carboplatin treatment. Consistent with our findings, a recent study has shown increased migratory, amino acid metabolism, protein catabolism and IFN1 signaling perturbation in platinum resistant ovarian cancer cell lines [60].

EMT in cancer cells has been associated with mitigating the immune system escape mechanisms in host [61]. In breast cancer, induction of EMT by overexpression of transcription factor SNAIL can render breast cancer cells resistant to the cytotoxic effect of CD8^+^T cells through the induction of autophagy; and targeting an autophagy inducer (BECN1) restored CD8^+^T mediated tumor cell lysis [62]. A recent pan cancer study indicated TGFBI as a prognostic marker in various cancers due to its involvement in various immune responses [63]. This is consistent with our analysis which showed a statistically significant positive association of infiltration of CD8^+^ T cells, CD4^+^ T cells, macrophages, neutrophils, and dendritic cells with TGFB1 expression. In addition, the enhanced expression of FLNA and ITGAV significantly associated with CD8^+^T cells, macrophages, and dendritic cells. Infiltration of dendritic cells was positively regulated by all the genes, except FLNA. As most of the ECM components in the study facilitated the infiltration of dendritic cells, may indicate essential role of remodeled ECM components in facilitating dendritic cell function which contributes in a significant way to antitumor response. However, approaches to use immunotherapy have not shown success in primary HGSOC or platinum resistant HGSOC patients [64, 65]. Potential reasons for the lack of immunotherapy response in HGSOC patients may include abundance of immunosuppressive factors in ovarian TME such as infiltration of a variety of immunosuppressive cells such as myeloid-derived suppressor cells (MDSC), cancer-associated fibroblasts (CAFs), tumor-associated macrophages (TAM) and Tregs. In addition, immunoregulatory enzymes (arginase, COX2, INOS) and immunosuppressive substances produced by these cells such as IL-10, TGFβ, vascular endothelial growth factor, PGE2, or PD-L1 inhibit innate and adaptive immunities and dendritic cell maturation. In addition, increased expression of checkpoint inhibitors such as PD-L1, CD47, CD73, in chemo naïve ovarian cancer cells and chemotherapy treated ovarian cancer cells have been noted [66, 67]. In addition, the density of intraepithelial CD8^+^T cells was shown to inversely correlate with the expression of PD-L1 on tumor cells, suggesting that the expression of PD-L1 on tumor cells may result in the exclusion of CD8 + T-cell in the tumors [66]. Some of these factors may indicate limited activity of immune check point inhibitors in treating chemonaive and platinum resistant ovarian cancer patients [65].

**Fig. 13:**
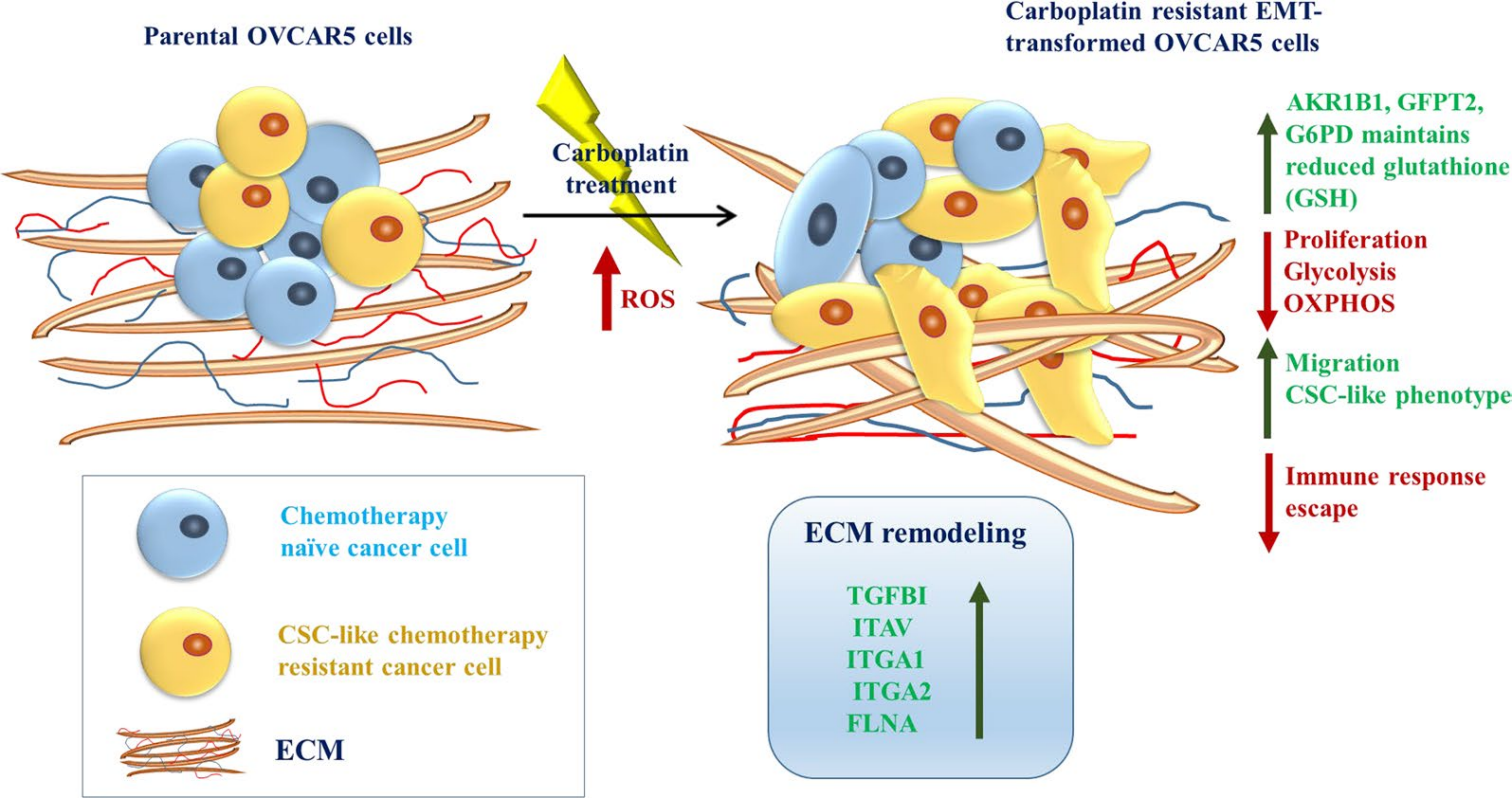
A model representing the mechanisms of survival, migration, and carboplatin resistance in OVCAR5 CBPR cell line deduced from the identification and validation of differential expressed proteins. Carboplatin resistant OVCAR5 cell line undergoes EMT transformation resulting in less proliferation and energy consumption but are more migratory in phenotype compared to parental cells. Altered metabolism in these cells results in upregulated G6PD expression which drives the pentose phosphate pathway, essential for reprogrammed metabolism and neutralization of increased oxidative stress (ROS) afflicted by carboplatin treatment through maintenance of GSH. Stabilization of ROS through sustenance of GSH is also provided by upregulation of AKR1B1 and GFPT2 expression. In addition, upregulation of TGFBI and related ECM components such as FLNA, ITGAV, ITGA1 and ITGA2 remodels the ECM to sustain chemoresistance and EMT transformation, essential for metastasis and survival of these cells

In summary, this study demonstrates that carboplatin-resistant cells acquired an EMT phenotype, which was less proliferative but more migratory. Altered expression of metabolic proteins also suggest a potential metabolic switch towards pentose phosphate pathway, polyol and hexosamine biosynthesis and purine salvage pathways with less dependency on anabolic gluconeogenesis and fatty acid biosynthesis. Enhanced expression of AKR1B1, GFPT2 and G6PD may sustain adequate level of GSH crucial for the survival of platinum—treated EMT transformed cells. On the other hand, upregulation of TGFBI in conjunction with ECM related proteins such as ITGAV, ITGA1, ITGA2, FLNA, etc. may facilitate ECM remodeling advantageous for chemoresistance and sustenance in the EMT transformed TME. Taken together, our findings provide molecular and functional evidence of EMT, altered metabolic and redox metabolism which supports carboplatin chemoresistance in ovarian cancer. The findings of this study have been depicted in Fig. 13.

## Supplementary Information

**Additional file 1: Figure S1.** Drug sensitivity of OVCAR5 parental and OVCAR5 resistance cells and the mRNA expression of drug resistant genes. (**A**) IC values for OVCAR5 CBPR cells and OVCAR5 parental cell lines by MTT assay. (**B**) mRNA expression of drug resistance geneABCG2 in OVCAR5 CBPR cells compared to OVCAR5 parental cells. mRNA expression was normalized to that of ACTB. n = 3; mean ± SD; student’s t test; **p < 0.01 when compared to OVCAR5 parental cells.

**Additional file 2: Table S1.** Details of primers used in qPCR validation of gene targets. **Table S2.** Clinical information on platinum-resistant, platinum-sensitive, initial diagnosed and relapsed patients. **Table S3.** (A): Peptide profile of proteins upregulated in OVCAR5CBPR compared to OVCAR5 cell line. **Table S3.** (B) Peptide profile of proteins downregulated in OVCAR5CBPR compared to OVCAR5 cell lines.

## Data Availability

The datasets used and/or analyzed during the current study are available from the corresponding author on reasonable request.

## Abbreviations

EMT: Epithelial mesenchymal transition
TME: Tumor microenvironment
TIMER: Tumor Immune Estimation Resource
HGSOC: High grade serous ovarian carcinoma
EOC: Epithelial ovarian cancer
MET: Mesenchymal epithelial transition
PBS: Phosphate buffered saline
BSA: Bovine serum albumin
OCR: Oxygen consumption rate
OXPHOS: Oxidative phosphorylation
ECAR: Extracellular acidification rate
2-DG: 2 Deoxy glucose
TCA: Tricarboxylic acid
ECM: Extracellular matrix
CTC: Circulating tumor cells
G6PD: Glucose-6-phosphate dehydrogenase
PPP: Pentose phosphate pathway
ITGA2: Integrin alpha-2
ITGAV: Integrin alpha-V
ITGA1: Integrin alpha-1
FLNA1: Filamin-A
TGFBI: Transforming growth factor induced protein
AKR1B1: Aldoreductase
GFPT2: Glutamine–fructose-6-phosphate aminotransferase
HPRT1: Hypoxanthine–guanine phosphoribosyltransferase
SDPR: Serum deprivation-response protein
CALB2: Calretinin
ALCAM: CD166 antigen
KTN1: Kinectin
SEPT2: Septin-6
FSCN1: Fascin
FLNC1: Filamin-C
PCK2: Phosphoenopyruvate carboxykinase
ACACA: Acetyl-CoA carboxylase 1; biotin carboxylase
MDSC: Myeloid-derived suppressor cells
CAFs: Cancer-associated fibroblasts
TAM: Tumor-associated macrophages
PD-L1: Programmed death-ligand 1
PGE2: Prostaglandin E2
GSH: Glutathione
