# Supplementary figures and images for: Platinum-resistance in epithelial ovarian cancer: an interplay of epithelial–mesenchymal transition interlinked with reprogrammed metabolism
Source: J Transl Med. 2022 Dec 3;20:556. doi: 10.1186/s12967-022-03776-y (PMC9719259; doi:10.1186/s12967-022-03776-y)

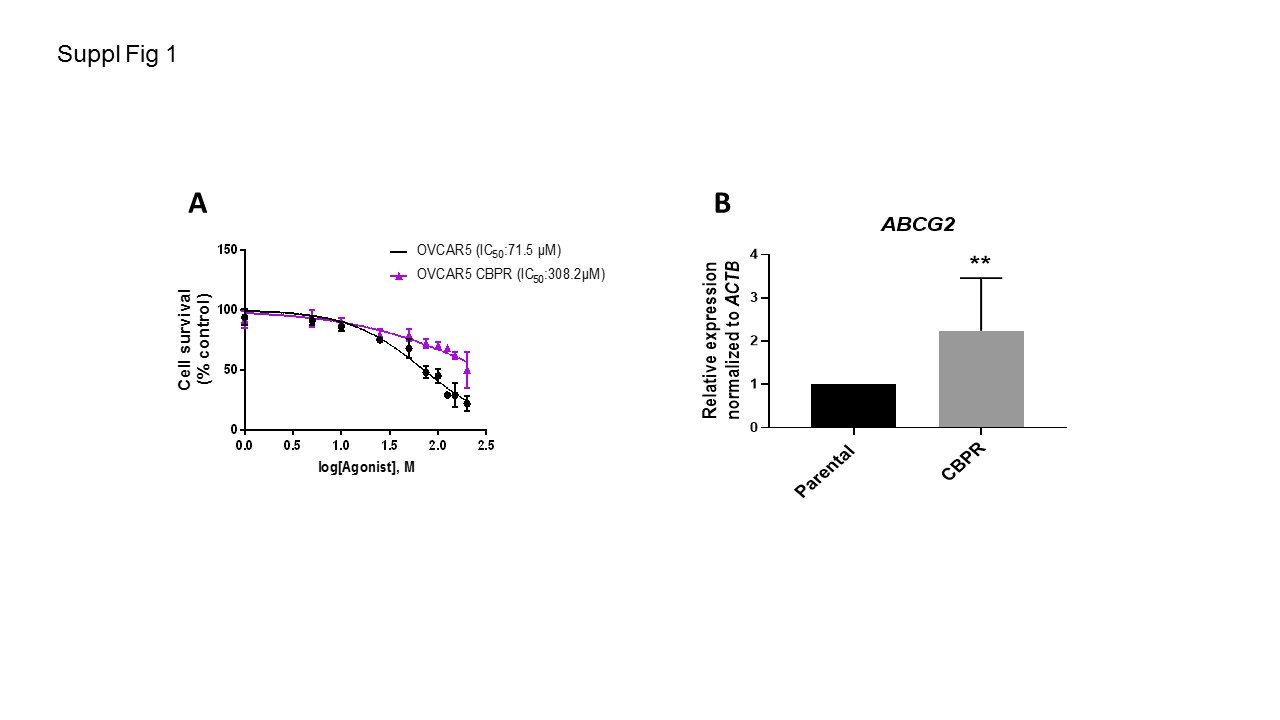

Supplement: Supplementary file 1 — Additional file 1: Figure S1. Drug sensitivity of OVCAR5 parental and OVCAR5 resistance cells and the mRNA expression of drug resistant genes. (A) IC values for OVCAR5 CBPR cells and OVCAR5 parental cell lines by MTT assay. (B) mRNA expression of drug resistance geneABCG2 in OVCAR5 CBPR cells compared to OVCAR5 parental cells. mRNA expression was normalized to that of ACTB. n = 3; mean ± SD; student’s t test; **p < 0.01 when compared to OVCAR5 parental cells. [file 12967_2022_3776_MOESM1_ESM.jpg]
