# Supplementary material for: Platinum-resistance in epithelial ovarian cancer: an interplay of epithelial–mesenchymal transition interlinked with reprogrammed metabolism
Source: J Transl Med. 2022 Dec 3;20:556. doi: 10.1186/s12967-022-03776-y (PMC9719259; doi:10.1186/s12967-022-03776-y)
Supplement: Supplementary file 2 — Additional file 2: Table S1. Details of primers used in qPCR validation of gene targets. Table S2. Clinical information on platinum-resistant, platinum-sensitive, initial diagnosed and relapsed patients. Table S3. (A): Peptide profile of proteins upregulated in OVCAR5CBPR compared to OVCAR5 cell line. Table S3. (B) Peptide profile of proteins downregulated in OVCAR5CBPR compared to OVCAR5 cell lines. [file 12967_2022_3776_MOESM2_ESM.docx]

**Table S1. Details of primers used in qPCR validation of gene targets.**

| Gene name | Accession ID | Forward (5'-3') | Reverse (5'-3') | Amplicon size (bp) | Annealing temperature (°C) |
| --- | --- | --- | --- | --- | --- |
| 18S | NR_003286.4 | GTAACCCGTTGAACCCCATT | CCATCCAATCGGTAGTAGCG | 150 | 60 |
| AKR1B1 | NM_001628.4 | CTCCAACTTCAACCATCTCCAG | ACCACGATGCCTTTGGACT | 143 | 58 |
| FLNA | NM_001456.3 | GCATCCAAAGTGGCACCAC | AGCCTCATAAGGGATGTACTCG | 166 | 60 |
| GFPT2 | NM_005110.4 | GCTCATCGTGATTGGCTGT | CCATCACAGGAAGCTCAGTC | 88 | 62 |
| ITGA1 | NM_181501.2 | CAGACGCTCAGTGGAGAACA | TGTACATAGGGGCTCCGACT | 111 | 60 |
| ITGA2 | NM_002203.4 | ATCATTCTCCCTGCCGGTT | GCTTGGAAACTGAGAGACGC | 179 | 60 |
| ITGAV | NM_002210.5 | AGGAGAAGGTGCCTACGAAG | GCACAGGAAAGTCTTGCTAAGG | 104 | 60 |
| SNAI1 | NM_005985.4 | GGTTCTTCTGCGCTACTGCT | TAGGGCTGCTGGAAGGTAAA | 146 | 60 |
| SQSTM1 | NM_003900.5 | GGTGCACCCCAATGTGATC | TCGCAGACGCTACACAAGTC | 101 | 60 |
| TGFBI | NM_000358.3 | TAACGGCCAGTACACGCTT | TGTTCAGCAGGTCTCTCAGG | 112 | 62 |

**Table S2. Clinical information on platinum-resistant, platinum-sensitive, initial diagnosed and relapsed patients.**

| **Patient** | **Diagnosis** | **Age at diagnosis (years)** | **Stage at diagnosis** | **Grade** | **Platinum-sensitive/ resistant** | **Progression-free survival (months)** |
| --- | --- | --- | --- | --- | --- | --- |
| 1 | Serous ovarian carcinoma | 66 | 3C | 3 | resistant | 8.5 |
| 2 | Serous ovarian carcinoma | 59 | 3C | 3 | sensitive | 13.6 |
| 3 | Serous ovarian carcinoma and tubal cancer | 69 | 3C | 3 | sensitive | 24.9 |
| 4 | Serous ovarian carcinoma | 62 | 3C | 3 | resistant | 7.2 |
| 5 | Serous ovarian carcinoma | 62 | 1C | 3 | sensitive | 46.1 |
| 6 | Serous ovarian carcinoma | 84 | 2A | 2 | resistant | 8.7 |
| 7 | Serous ovarian carcinoma | 81 | 3 | 2 | resistant | 4.1 |
| 8 | Serous ovarian carcinoma | 44 | 3C | 3 | sensitive | 12.9 |
| 9 | Serous ovarian carcinoma | 61 | 3C | 3 | sensitive | 13.4 |
| 10 | Peritoneal cancer | 43 | 3C | 3 | sensitive | 28.6 |
| 11 | Serous papillary carcinoma of the ovary | 75 | 3C | 3 | sensitive | 23.8 |
| 12 | Serous papillary carcinoma of the ovary | 46 | 3 | 3 | sensitive | 51.0 |
| 13 | Serous papillary carcinoma of the ovary | 42 | 3c | 3 | sensitive | 37.4 |
| 14 | Serous papillary carcinoma of the ovary | 42 | 3c | 3 | sensitive | 41.0 |
| 15 | Serous carcinoma of gynaecologi-cal origin | 63 | 3c | 3 | resistant | 13.1 |
| 16 | Serous papillary carcinoma of the ovary | 60 | 3c | 3 | sensitive | 16.5 |
| 17 | Serous papillary carcinoma of the ovary | 78 | 3a | 3 | resistant | 9.6 |
| 18 | Serous papillary carcinoma of the ovary | 46 | 3c | 3 | resistant | 8.5 |
| 19 | Serous carcinoma of the ovary | 61 | 3c | 3 | sensitive | 35.3 |
| 20 | Serous carcinoma of the peritoneum | 48 | 3a | 3 | resistant | 0.0 |
| 21 | Serous papillary carcinoma of the ovary | 66 | 3c | 3 | sensitive | 23.1 |
| 22 | Serous papillary carcinoma of the ovary | 63 | 2c | 3 | resistant | 10.2 |
| 23 | Serous papillary carcinoma of the ovary | 52 | 3c | 3 | resistant | 8.8 |
| 24 | Serous carcinoma of the ovary | 60 | 2a | 3 | sensitive | NA |
| 25 | High grade serous carcinoma of the ovary | 66 | 4 | 3 | sensitive | 8.4 |

Platinum-sensitive: n=15

Platinum-resistant: n=10

**Matching relapsed samples-**

**At Diagnosis**  **Relapsed**

5 26

12 27

14 28

17 29

**Table S3 (A): Peptide profile of proteins upregulated in OVCAR5CBPR compared to OVCAR5 cell line.**

| Accession ID | Gene names | Protein names | Peptide sequence | Sequence coverage | Protein score | Fold change (CBPR/Control) | p value |
| --- | --- | --- | --- | --- | --- | --- | --- |
| P32455 | GBP1 | Interferon-induced guanylate-binding protein 1 | EKEIEVER ESMTDAILQTDQTLTEK GFSLGSTVQSHTK SSPDENENEVEDSADFVSFFPDFVWTLR AIAHYEQQMGQK DVDHLFQK | 14.5 | 37.293 | 3.92 | 0.0011 |
| P11413 | G6PD | Glucose-6-phosphate 1-dehydrogenase | CISEVQANNVVLGQYVGNPDGEGEATK DGLLPENTFIVGYAR DNIACVILTFK DVMQNHLLQMLCLVAMEKPASTNSDDVRDEK EELFQGDAFHQSDTHIFIIMGASGDLAK EMVQNLMVLR GGYFDEFGIIR GPTEADELMK GSTTATFAAVVLYVENER GYLDDPTVPR IDHYLGK IFGPIWNR IFTPLLHQIELEK  KPGMFFNPEESELDLTYGNR LEDFFAR LFYLALPPTVYEAVTK LILDVFCGSQMHFVR LNSHMNALHLGSQANR LPDAYER LQFHDVAGDIFHQQCK LSNHISSLFR LTVADIR NIHESCMSQIGWNR NSYVAGQYDDAASYQR QSEPFFK RVGFQYEGTYK TQVCGILR VGFQYEGTYK WDGVPFILR | 73.4 | 184.37 | 3.64 | 0.0002 |
| P17301 | ITGA2 | Integrin alpha-2 | AGDISCNADINPLK CPVDLSTATCEK DCGEDGLCISDLVLDVR FGIAVLGYLNR FVQGLDIGPTK IGQTSSSVSFK IPLLYDAEIHLTR SVACDVGYPALK TSHGHLIFPK VDISLENPGTSPALEAYSETAK | 11.3 | 80.444 | 3.61 | 0.0001 |
| Q16555 | DPYSL2 | Dihydropyrimidinase-related protein 2 | AITIANQTNCPLYITK FQLTDCQIYEVLSVIR FQMPDQGMTSADDFFQGTK GIQEEMEALVK GLYDGPVCEVSVTPK GTVVYGEPITASLGTDGSHYWSK IAVGSDADLVIWDPDSVK IVLEDGTLHVTEGSGR KGTVVYGEPITASLGTDGSHYWSK MVIPGGIDVHTR THNSSLEYNIFEGMECR VFNLYPR MDENQFVAVTSTNAAK QIGENLIVPGGVK | 35 | 64.638 | 3.58 | 0.0005 |
| Q12913 | PTPRJ | Receptor-type tyrosine-protein phosphatase eta | AVSISPTNVILTWK DFIATQGPLPNTLK GASDTYVTYLIR GDPLGTEGGLDASNTER GLIDGAESYVSFSR NNEVSFSQIKPK SPDGASEYVYHLVIESK VENFEAYFK VITEPIPVSDLR | 9.1 | 30.007 | 3.24 | 0.0208 |
| Q15582 | TGFBI | Transforming growth factor-beta-induced protein ig-h3 | DLLNNHILK EGVYTVFAPTNEAFR GDELADSALEIFK IPSETLNR  LTLLAPLNSVFK NSLCIENSCIAAHDK QAGLGNHLSGSER QHGPNVCAVQK SPYQLVLQHSR STVISYECCPGYEK VISTITNNIQQIIEIEDTFETLR VLTPPMGTVMDVLK YGTLFTMDRYLYHGQTLETLGGK | 26.5 | 89.138 | 3.13 | 0.0135 |
| Q15738 | NSDHL | Sterol-4-alpha-carboxylate 3-dehydrogenase, decarboxylating | CTVIGGSGFLGQHMVEQLLAR DPQLVPILIEAAR FFLGDLCSR GVNTVFHCASPPPSSNNK GYAVNVFDIQQGFDNPQVR ILTGLNYEAPK THLTEDTPK VALAGTFHYYSCER VNADIEK | 32.4 | 63.853 | 3.05 | 0.0005 |
| P15121 | AKR1B1 | Aldose reductase | DYPFHEEF GIVVTAYSPLGSPDRPWAK GIVVTAYSPLGSPDRPWAKPEDPSLLEDPR HIDCAHVYQNENEVGVAIQEK LDYLDLYLIHWPTGFKPGK LIQYCQSK LLLNNGAK LWCTYHEK NLVVIPK PAVNQIECHPYLTQEK SPPGQVTEAVK VAIDVGYR VCALLSCTSHK VFDFELSSQDMTTLLSYNR YKPAVNQIECHPYLTQEK TTAQVLIR | 58.2 | 105.9 | 3.00 | 0.0017 |
| P51572 | BCAP31 | B-cell receptor-associated protein 31 | AENQVLAMR KYMEENDQLK KYMEENDQLKK LDVGNAEVK LLEEHAK LQAAVDGPMDK LVTLISQQATLLASNEAFK VNLQNNPGAMEHFHMK YMEENDQLKK | 33.3 | 44.299 | 2.89 | <0.0001 |
| Q13642 | FHL1 | Four and a half LIM domains protein 1 | AITSGGITYQDQPWHADCFVCVTCSK AIVAGDQNVEYK CLHPLANETFVAK DCFTCSNCK FCANTCVECR FDCHYCR FTAVEDQYYCVDCYK FWHDTCFR GEDFYCVTCHETK QVIGTGSFFPK | 38.4 | 128.24 | 2.59 | 0.0227 |
| P06756 | ITGAV | Integrin alpha-V;Integrin alpha-V heavy chain;Integrin alpha-V light chain | AGTQLLAGLR DLALSEGDIHTLGCGVAQCLK EPVGTCFLQDGTK EQLQPHENGEGNSET GGLMQCEELIAYLR ILACAPLYHWR IYIGDDNPLTLIVK MFLLVGAPK QAHILLDCGEDNVCKPK SAILYVK SHQWFGASVR STGLNAVPSQILEGQWAAR TVEYAPCR VRPPQEEQER | 17 | 35.81 | 2.52 | 0.0014 |
| P00492 | HPRT1 | Hypoxanthine-guanine phosphoribosyltransferase | DLNHVCVISETGK FFADLLDYIK FVVGYALDYNEYFR NVLIVEDIIDTGK SIPMTVDFIR SVGYKPDFVGFEIPDK SYCNDQSTGDIK TMQTLLSLVR VFIPHGLIMDR VIGGDDLSTLTGK VASLLVK | 59.2 | 62.136 | 2.48 | 0.0008 |
| Q13636 | RAB31 | Ras-related protein Rab-31 | EYAESIGAIVVETSAK FVQDHFDHNISPTIGASFMTK GSAAAVIVYDITK NAINIEELFQGISR FLIWDTAGQER VCLLGDTGVGK | 44.3 | 16.503 | 2.45 | 0.0146 |
| Q13501 | SQSTM1 | Sequestosome-1 | AGEARPGPTAESASGPSEDPSVNFLK CSVCPDYDLCSVCEGK DHRPPCAQEAPR EAALYPHLPPEADPR FSFCCSPEPEAEAEAAAGPGPCER IALESEGRPEEQMESDNCSGGDDDWTHLSSK LAFPSPFGHLSEGFSHSR LIESLSQMLSMGFSDEGGWLTR LTPVSPESSSTEEK NMVHPNVICDGCNGPVVGTR NVGESVAAALSPLGIEVDIDVEHGGK NYDIGAALDTIQYSK PGPTAESASGPSEDPSVNFLK RFSFCCSPEPEAEAEAAAGPGPCER SSSQPSSCCSDPSKPGGNVEGATQSLAEQMR VAALFPALR | 63.6 | 157.11 | 2.12 | 0.0293 |
| P56199 | ITGA1 | Integrin alpha-1 | EDTIYEADLQYR FEYQMSLEPIK TIVHYSPNLVFSGIEAIQK VIQDCEDENIQR VMVIVTDGESHDNHR YSSTEEVLVAAK | 6.9 | 27.575 | 2.10 | 0.0478 |
| O94808 | GFPT2 | Glutamine--fructose-6-phosphate aminotransferase [isomerizing] 2 | EITYMHSEGILAGELK ETDCGVHINAGPEIGVASTK GSPLLIGVR GYDVDFPR QVLEELTELPVMVELASDFLDR CQNALQQVTAR EIFEQPESVFNTMR EIFETLIK ETEDITFSTLVER GYDSAGVAIDGNNHEVK GYEFESETDTETIAK GYNYATCLEGALK LSTEQIPILYR WATHGVPSAVNSHPQR | 28.3 | 53.837 | 2.07 | 0.0086 |
| P07099 | EPHX1 | Epoxide hydrolase 1 | DVELLYPVK EDDSIRPFK GGHFAAFEEPELLAQDIR IEGLDIHFIHVKPPQLPAGHTPK NHGLSDEHVFEVICPSIPGYGFSEASSK VETSDEEIHDLHQR YLEDGGLER | 24.2 | 24.912 | 2.02 | 0.0216 |
| P21333 | FLNA | Filamin-A | AEAGVPAEFSIWTR AEGPGLSR AEISFEDR AFGPGLQGGSAGSPAR AGNNMLLVGVHGPR AGQSAAGAAPGGGVDTR AHEPTYFTVDCAEAGQGDVSIGIK ALGALVDSCAPGLCPDWDSWDASKPVTNAR ALTQTGGPHVK ANLPQSFQVDTSK APSVANVGSHCDLSLK ASGPGLNTTGVPASLPVEFTIDAK ATCAPQHGAPGPGPADASK AYGPGIEPTGNMVK CAPGVVGPAEADIDFDIIR CSGPGLSPGMVR CSYQPTMEGVHTVHVTFAGVPIPR DAGEGGLSLAIEGPSK DAGEGLLAVQITDPEGK DAGEGLLAVQITDPEGKPK DAPQDFHPDR DGSCGVAYVVQEPGDYEVSVK DGSCSVEYIPYEAGTYSLNVTYGGHQVPGSPFK DKGEYTLVVK DNGNGTYSCSYVPR DQEFTVK DVDIIDHHDNTYTVK EAGAGGLAIAVEGPSK EAMQQADDWLGIPQVITPEEIVDPNVDEHSVMTYLSQFPK EATTEFSVDAR EEGPYEVEVTYDGVPVPGSPFPLEAVAPTKPSK EGPYSISVLYGDEEVPR ENGVYLIDVK ETGEHLVHVK FGGEHVPNSPFQVTALAGDQPSVQPPLR FNEEHIPDSPFVVPVASPSGDAR FNGTHIPGSPFK FVPAEMGTHTVSVK GAGSYTIMVLFADQATPTSPIR GAGTGGLGLAVEGPSEAK GEYTLVVK GLVEPVDVVDNADGTQTVNYVPSR GTVEPQLEAR HTAMVSWGGVSIPNSPFR IANLQTDLSDGLR IPEISIQDMTAQVTSPSGK IVGPSGAAVPCK KDGSCGVAYVVQEPGDYEVSVK LDVQFSGLTK LPQLPITNFSR LQVEPAVDTSGVQCYGPGIEGQGVFR LTVSSLQESGLK LVSNHSLHETSSVFVDSLTK LYSVSYLLK MDCQECPEGYR NGHVGISFVPK RAEFTVETR RAPSVANVGSHCDLSLK RLTVSSLQESGLK SADFVVEAIGDDVGTLGFSVEGPSQAK SAGQGEVLVYVEDPAGHQEEAK SPFEVYVDK SPFSVAVSPSLDLSK SPYTVTVGQACNPSACR SSFTVDCSK TFSVWYVPEVTGTHK TGVAVNKPAEFTVDAK TGVELGKPTHFTVNAK THIQDNHDGTYTVAYVPDVTGR TPCEEILVK VANPSGNLTETYVQDR VAQPTITDNK VAQPTITDNKDGTVTVR VDVGKDQEFTVK VEPGLGADNSVVR VGSAADIPINISETDLSLLTATVVPPSGR VHGPGIQSGTTNKPNK VHSPSGALEECYVTEIDQDK VHSPSGALEECYVTEIDQDKYAVR VLPTHDASK VNQPASFAVSLNGAK VNVGAGSHPNK VPVHDVTDASK VQVQDNEGCPVEALVK VTAQGPGLEPSGNIANK VTVLFAGQHIAK VTYTPMAPGSYLISIK VYGPGVAK YAPSEAGLHEMDIR YGGDEIPFSPYR YGGPYHIGGSPFK YGGQPVPNFPSK YNEQHVPGSPFTAR YTPVQQGPVGVNVTYGGDPIPK YWPQEAGEYAVHVLCNSEDIR QMQLENVSVALEFLDR DLAEDAPWK DLAEDAPWKK IQQNTFTR LIALLEVLSQK LLGWIQNK LVSIDSK WCNEHLK GQHVPGSPFQFTVGPLGEGGAHK IECDDKGDGSCDVR NDNDTFTVK | 57.9 | 323.31 | 2.01 | 0.0028 |

**Table S3 (B): Peptide profile of proteins downregulated in OVCAR5CBPR compared to OVCAR5 cell lines.**

| Accession ID | Gene names | Protein names | Peptide sequence | Sequence coverage | Protein score | Fold change (CBPR/Control) | p value |
| --- | --- | --- | --- | --- | --- | --- | --- |
| O95810 | SDPR | Serum deprivation-response protein | DNSQVNAVTVLTLLDK GIQNDLTK LENNHAQLLR LVNMLDAVQENQHK RLENNHAQLLR SDGDPVQPAVLQVHQTS VLIFQEENEIPASVFVK YEGSYALTSEEAER YQASTSNTVSK | 25.4 | 104.96 | 7.10 | 0.0172 |
| P22676 | CALB2 | Calretinin | AGPQQQPPYLHLAELTASQFLEIWK ELENFFQELEK GFLSDLLK HFDADGNGYIEGK LLPVQENFLLK LQEYTQTILR SGYIDEHELDALLK | 33.9 | 37.604 | 5.76 | 0.0033 |
| Q16822 | PCK2 | Phosphoenolpyruvate carboxykinase [GTP], mitochondrial | AIDTTQLFSLPK CLHSVGQPLTGQGEPVSQWPCNPEK EGALDLSGLR ETPIGLVPK EVLAELEALER GQLGNWMSPADFQR GVPLVYEAFNWR LCQPEGIHICDGTEAENTATLTLLEQQGLIR LGTPVLQALGDGDFVK SYLTEQVNQDLPK VECVGDDIAWMR VLDWICR VLSGDLGQLPTGIR EIISFGSGYGGNSLLGK | 31.7 | 67.532 | 4.78 | 0.0403 |
| Q13740 | ALCAM | CD166 antigen | ADIQMPFTCSVTYYGPSGQK QIGDALPVSCTISASR SSNTYTLTDVR SSPSFSSLHYQDAGNYVCETALQEVEGLK SVQYDDVPEYK YEKPDGSPVFIAFR | 17.3 | 21.726 | 3.68 | 0.0216 |
| Q86UP2 | KTN1 | Kinectin | AAGDTTVIENSDVSPETESSEK AHVQEVAQHNLK DAVSNTTNQLESK DLLTELQK EEELKDIQNMNFLLK EEIGNVQLEK EHNVFQNK ENEVQSLHSK LLEEQLQHEISNK LQALANEQAAAAHELEK LQTLVSEQPNK QNDQVSFASLVEELKK QQQVEAVELEAK RQEALPLHQETK SVLAETEGILQK TAEHEAAQQDLQSK TQLLQDVQDENK WLQDLQEENESLK | 16.9 | 139.2 | 3.54 | 0.0399 |
| Q14141 | SEPT6 | Septin-6 | AATDIAR EDSYKPIVEFIDAQFEAYLQEELK FEGEPATHTQPGVQLQSNTYDLQESNVR LTIVSTVGFGDQINK SLDDEVNAFK TAAELLQSQGSQAGGSQTLK VLHTYHDSR | 40.1 | 38.387 | 3.44 | 0.0393 |
| Q16658 | FSCN1 | Fascin | DELFALEQSCAQVVLQAANER DVPWGVDSLITLAFQDQR FLIVAHDDGR GEHGFIGCR LINRPIIVFR LSCFAQTVSPAEK LVARPEPATGYTLEFR NASCYFDIEWR QIWTLEQPPDEAGSAAVCLR VTGTLDANR WSLQSEAHR YAHLSAR YFGGTEDR YLAADKDGNVTCER YLAPSGPSGTLK YLTAEAFGFK YSVQTADHR YWTLTATGGVQSTASSK | 45.2 | 164.69 | 2.66 | 0.0052 |
| Q13085 | ACACA | Acetyl-CoA carboxylase 1;Biotin carboxylase | ATLVDHGIR DIIVIGNDITYR DLAEWLEK DPSLPLLELQDIMTSVSGR DPTLTDELLNILTELTQLSK DVDDGLQAAEEVGYPVMIK ECCQPVLVYIPPQAELR EEGIGPENLR IDTGWLDR IGSFGPQEDLLFLR IIEEAPATIATPAVFEHMEQCAVK IIQQAGQVWFPDSAFK ILNVPQELYEK LGGIPVGVVAVETR LLETESFQMNR LLLEDLVK LPELLLK LTFLVAQK MMYGVSPWGDSPIDFEDSAHVPCPR RVDPVYIHLAER SVHSSVPLLNSK TDCDIEDDRLAAMFR VDWQENDFSK VEVGTEVTDYR VLQAELK VNNADDFPNLFR VSALNSVHCEHVEDEGESR YLYLTPQDYK DFTVASPAEFVTR EEAISNMVVALK FGAYIVDGLR ITSENPDEGFKPSSGTVQELNFR | 18.2 | 62.384 | 2.34 | 0.0003 |
| Q9NP81 | SARS2 | Serine--tRNA ligase, mitochondrial | DGSVLVPPALQSYLGTDR FCACPEEAAHALELR FDIEAWMPGR FGEVTSASNCTDFQSR ITAPTHVPLQYIGPNQPR LLIALLESNQQK LPNQTHPDVPVGDESQAR VLDMPTQELGLPAYR | 23.6 | 51.46 | 2.32 | 0.0059 |
| Q9Y570 | PPME1 | Protein phosphatase methylesterase 1 | DFSPVPWSQYFESMEDVEVENETGK DLTIGQMQGK FQMQVLPQCGHAVHEDAPDK GLSNLFLSCPIPK LDKDLTIGQMQGK LLLLAGVDR LPSRPPLPGSGGSQSGAK QCEGITSPEGSK SIVEGIIEEEEEDEEGSESISK SIVEGIIEEEEEDEEGSESISKR SLENAIEWSVK VAEAVATFLIR VKNPEDLSAETMAK VSMVGQVK YWDGWFR | 47.7 | 172.61 | 2.30 | 0.0118 |
| Q14315 | FLNC | Filamin-C | DLAEDAPWK DLAEDAPWKK IQQNTFTR LIALLEVLSQK LLGWIQNK LVSIDSK WCNEHLK GQHVPGSPFQFTVGPLGEGGAHK IECDDKGDGSCDVR NDNDTFTVK EAGAGGLSIAVEGPSK GAGTGGLGLTVEGPCEAK AEIAFEDR APLQVAVLGPTGVAEPVEVR APSGNEEPCLLK CSGPGLGAGVR DGSCGVSYVVQEPGDYEVSIK DNGDGTHTVHYTPATDGPYTVAVK EVGEHVVSVR FNDEHIPDSPFVVPVASLSDDAR GDYILIVK GGLVGTPAPFSIDTK GVAGVPAEFSIWTR LGSFGSITR LSGGHSLHETSTVLVETVTK  QAPSIATIGSTCDLNLK TPCEEVYVK TSQLNVGTSTDVSLK VAVGQEQAFSVNTR VCAYGPGLK VDITYDGHPVPGSPFAVEGVLPPDPSK VEAAEIVEGEDSAYSVR VEESTQVGGDPFPAVFGDFLGR VHTPSGAVEECYVSELDSDK VHTPSGAVEECYVSELDSDKHTIR VYNVTYTVK | 17.9 | 61.61 | 2.25 | 0.0192 |
| P13726 | F3 | Tissue factor | CFYTTDTECDLTDEIVK DLIYTLYYWK GENYCFSVQAVIPSR STDSPVECMGQEK TILEWEPKPVNQVYTVQISTK TNTNEFLIDVDK | 29.8 | 140.55 | 2.20 | 0.0359 |
| Q15084 | PDIA6 | Protein disulfide-isomerase A6 | ALDLFSDNAPPPELLEIINEDIAK DGELPVEDDIDLSDVELDDLGKDEL DVIELTDDSFDK GESPVDYDGGR GSTAPVGGGAFPTIVER GSTAPVGGGAFPTIVEREPWDGR HHSLGGQYGVQGFPTIK KDVIELTDDSFDK LAAVDATVNQVLASR NLEPEWAAAASEVK NSYLEVLLK TCEEHQLCVVAVLPHILDTGAAGR TGEAIVDAALSALR | 43 | 96.534 | 2.13 | 0.047 |
| P24666 | ACP1 | Low molecular weight phosphotyrosine protein phosphatase | EDFATFDYILCMDESNLR IELLGSYDPQK LVTDQNISENWR SPIAEAVFR SVLFVCLGNICR VDSAATSGYEIGNPPDYR | 50.6 | 18.531 | 2.11 | 0.0112 |
